# Xerogel-Derived Manganese Oxide/N-Doped Carbon as a Non-Precious Metal-Based Oxygen Reduction Reaction Catalyst in Microbial Fuel Cells for Energy Conversion Applications

**DOI:** 10.3390/nano13222949

**Published:** 2023-11-15

**Authors:** Wu Hao, Sang-Hun Lee, Shaik Gouse Peera

**Affiliations:** Department of Environmental Science, Keimyung University, Daegu 42601, Republic of Korea

**Keywords:** xerogel, MnO_2_, coffee carbon, N-doped carbon, ORR, cathode catalyst, microbial fuel cells (MFCs)

## Abstract

Current study provides a novel strategy to synthesize the nano-sized MnO nanoparticles from the quick, ascendable, sol-gel synthesis strategy. The MnO nanoparticles are supported on nitrogen-doped carbon derived from the cheap sustainable source. The resulting MnO/N-doped carbon catalysts developed in this study are systematically evaluated via several physicochemical and electrochemical characterizations. The physicochemical characterizations confirms that the crystalline MnO nanoparticles are successfully synthesized and are supported on N-doped carbons, ascertained from the X-ray diffraction and transmission electron microscopic studies. In addition, the developed MnO/N-doped carbon catalyst was also found to have adequate surface area and porosity, similar to the traditional Pt/C catalyst. Detailed investigations on the effect of the nitrogen precursor, heat treatment temperature, and N-doped carbon support on the ORR activity is established in 0.1 M of HClO_4_. It was found that the MnO/N-doped carbon catalysts showed enhanced ORR activity with a half-wave potential of 0.69 V vs. RHE, with nearly four electron transfers and excellent stability with just a loss of 10 mV after 20,000 potential cycles. When analyzed as an ORR catalyst in dual-chamber microbial fuel cells (DCMFC) with Nafion 117 membrane as the electrolyte, the MnO/N-doped carbon catalyst exhibited a volumetric power density of ~45 mW m^2^ and a 60% degradation of organic matter in 30 days of continuous operation.

## 1. Introduction

Microbial fuel cells (MFC) are considered futuristic energy generation technology that simultaneously treat wastewater and produce useful energy via combined biochemical and electro-chemical processes. MFC uses the biological and catalytic process to extract energy and degrade the organic pollutants from wastewater [1,2]. MFC has been widely investigated to treat wastewater from various sources [2]. Despite significant developments in the MFC technology, the commercialization of the MFC is stalled by several factors such as the slow anodic bacterial catabolism of organic pollutants [3], slow kinetics of the cathodic ORR catalysts [4], high cost of the Pt-based catalysts used in cathodes [5], high cost of the Nafion membrane [6], low power output [7], low treatment efficiency [8], and practical difficulties in implementing the MFCs in real wastewater treatment plants [9]. Among these problems, the cathodic ORR is considered as one of the major obstacles both in terms of its slow, sluggish kinetics and poor stability of the cathode catalyst [10]. In general, Pt/C has been used as an ORR catalyst; however, due to its high cost, limited reserves, and its weakness towards poisoning and deactivation in the presence of typical MFC metabolites, it led us to search for alternative catalysts that are cost effective and, at the same time, as efficient as the Pt/C catalyst [11]. Several researchers explored various transition metal-based catalysts composed of Co and Fe [12,13]. Several excellent catalysts of the Fe-N-C/Co-N-C catalysts have been proposed in the past literature [14,15,16,17]. For instance, the atomically dispersed Fe-N-C catalysts by the metal–organic framework catalysts [18,19]. Though excellent catalysts have been proposed, the synthesis of such catalysts would require specific organic precursors such as 2-methyl imidazole and many other organic ligands such as terephthalic acid and isophthalic acid, trimesic acid, 4,4′-bipyridine and pyrazine, 1,2,3-triazole derivatives, 1,3,5-benzenetriamine, etc. All of these are required in their purest form, which would increase the catalyst cost from the precursor’s perspectives, in addition to the requirement of the Zn metal nodes [20,21]. Despite the admirable ORR activity, the Fe-N-C type of catalysts suffer from the poor durability, due to the fact that the Fe-based catalysts catalyzes the Fenton type reactions that produce H_2_O_2_ as a byproduct that could degrade the catalyst and the Nafion membrane, whereas the Co-based catalyst also produces a considerable 2 + 2 O_2_ reduction pathway together with low ORR kinetics [22,23]. In this regard, exploring non-Fenton reactive metals, such as Mn and Cu, is considered as the best choice to mitigate the catalyst degradation via Fenton-associated reactions; therefore, to increase the overall catalyst stability [14].

Among the non-Pt metal-based catalysts, the transition metal–oxides as the ORR catalysts in MFCs, in particular manganese oxides (MnO_2_), have been widely investigated, majorly due to the low cost and high chemical and electrochemical stability [24]. Several researchers explored various methods to synthesize MnO_2_ such as the chemical oxidation of MnSO_4_, hydrothermal methods, electrochemical deposition of MnO_2_, thermal decomposition of KMnO_4_, and co-precipitation, microwave heating, and electrodeposition methods [17,18,24,25,26]. In this study, we synthesized the MnO_2_ via a simple sol-gel method, where the obtained gel is dried in a hot-air oven to form a manganese oxide xerogel, which was further pyrolyzed to obtain the MnO nanoparticles [27,28,29]. Although promising as the ORR catalysts, MnO_x_’s intrinsic electrocatalytic ORR activity is limited by its low electrical conductivity, ease of agglomeration, and low chemical stability under electrochemical operational conditions [29]. To resolve these issues, it is common practice to use highly conductive materials as a support for MnO_2_, such as graphene, graphene-oxide, carbon black, single/multiwalled carbon nanotubes, and activated carbons [30]. Conducting carbon supports have been known to provide sufficient surface area to anchor the metal–oxide nanoparticles. The carbon support helps in effective electron transport from the support to the ORR active sites and enhances the stability of the metal–oxide particles via strong metal–support interactions [31]. Furthermore, doping/chemical functionalization of the carbon supports, especially via nitrogen doping, is found to improve the adsorption of oxygen and the decomposition of peroxide intermediates during the ORR [32]. Finally, the N-doped carbons itself has proved to be a catalytic material for ORR [33]. Therefore, the use of N-doped carbons as a support for the MnO_x_ nanoparticles could bring several advantages such as (i) a synergistic co-catalyst for ORR serving as ORR catalytic centers (ii) acting as a stable catalyst support for anchoring and dispersing the MnO_2_ evenly without agglomerations, which (iii) improves the strength of MnO_2_ and the carbon support by acting as the N-doped locations’ tapping sites, which could prevent the dissolution/agglomeration of MnO_2_ under acidic electrolytic conditions, thus improving the overall durability of the catalyst [34].

In this regard, several carbon materials such as carbon black, activated carbons, carbon nanotubes, carbon nanofibers, graphene, etc., have been used as a substrate for the N doping [35,36,37,38]. Recently, exploring the renewable, low cost, wasted biomass as a substrate to synthesize the catalyst materials is gaining tremendous interest, due to the fact that the accumulation of biomass waste on the planet earth is increasingly becoming a serious problem [37,38]. Therefore, several researchers examined the possibility of using low-cost biomass waste from different sources to make carbon materials for several applications including the catalyst support for electrochemical reactions such as ORR and several other electrochemical applications [39,40,41,42,43,44,45]. Often, the carbon resulting from the pyrolysis of the biomass shows a high surface area, porosity, good graphitization, and balanced hydrophilic/hydrophobicity and electronic conductivity similar to the traditional carbons, which makes it a good place to add heteroatoms to boost the activity of the catalyst [43]. Among various types of biomasses, coffee waste is particularly interesting. When used in the beverage industry, about 0.9 kg of coffee waste is produced for every 1 kg of raw coffee ground used. This led to millions of tons of coffee waste being generated and thrown away annually. When this coffee waste are put in landfill or remained in environment, it undergoes natural decomposition and releases methane (CH_4_) gas, which has GWP (Global Warming Potential) of 20 times higher than CO_2_ [44]. Moreover, the coffee carbon-derived ORR catalysts have also been reported in the literature [45,46,47,48,49,50,51] Therefore, it is very much convincing that waste coffee grounds could be a best choice to use as raw materials for the synthesis of porous carbon nanomaterials.

In this work, we used coffee waste collected from the local coffee vending machine, washed, purified, and then used as raw material for the synthesis of the porous carbon to be used as a catalyst support for the MnO nanoparticles. Further, a systematic relationship on the effect of N-doping to the coffee ground and the ORR activity in acidic electrolytes was established by varying the mass ratio of the waste coffee-derived carbons and the melamine as nitrogen precursors. The optimum content of N in the doped carbon was systematically investigated by rotating disk electrode (RDE) studies and the effect of N doping on the carbon structures was established via X-ray diffraction (XRD) analysis. The morphological observation of the optimized N-doped carbons was obtained via the scanning electron microscope (SEM) analysis. The optimized N-doped carbon was then used as a support to deposit the MnO nanoparticles via a simple sol-gel synthesis, followed via pyrolysis at a defined temperature of 900 °C. A systematic investigation on the effect of N-doped carbon and the loading of MnO was established via RDE studies in 0.1 M of HClO_4_ electrolyte. The optimized catalyst MnO/N-doped carbon was analyzed using various physiochemical characterizations such as X-ray diffraction (XRD), scanning electron microscopy (SEM), transmission electron microscopy (TEM), X-ray photoelectron spectroscopy (XPS) (X-ray photoelectron spectroscopy), Brunauer–Emmett–Teller (BET) analysis, and electrochemical characterization such as CV (cyclic voltammetry), and the LSV (linear sweep voltammetry) stability test in 0.1 M of HClO_4_ electrolyte. Furthermore, the optimized MnO/N-doped carbon catalyst has been used as a cathode catalyst and its performance was evaluated in dual-chamber microbial fuel cells, both in terms of volumetric power density as a function of cathodic ORR activity and the degradation of organic matter in the anodic chamber by measuring the TOC at specified intervals of times. After a detailed investigation, it was found that the MnO/N-doped carbon catalyst showed excellent ORR activity similar to the traditional, benchmark Pt/C catalyst in RDE studies and in MFC performance, together with the excellent stability of over 20,000 potential cycles in acidic electrolytes.

## 2. Materials and Methods

### 2.1. Carbon Synthesis Using Coffee Waste

After being used, waste coffee power was collected from the local coffee vending machine shop, which was then washed several times with the distilled water to remove any dissolvable impurities, and the resulting material was then dried in a hot-air oven at 80 °C and for 12 h. The resulting dried powder was then ground using a mortar and pestle and a defined mass was then subjected to pyrolysis in a tubular furnace at the temperature of 900 °C for 1 h at the heating rate of 5 °C/min, under a continuous flow of inert N_2_ gas. After the pyrolysis, a black carbon powder was obtained, which was ground again into fine power using a mortar and pestle and was used for further studies. The catalyst obtained in this way was designated as pyrolyzed carbon derived from the waste coffee grounds (P-CC).

### 2.2. N-Doped Carbon Synthesis Using Coffee Waste and Melamine

Melamine (C_3_H_6_N_6_) was used as an N precursor to synthesize N-doped carbon. The N-doping was performed both for raw-CC (without pyrolysis) and P-CC carbon materials. In a typical synthesis, 100 mg of the raw-CC and P-CC was dispersed in 50 mL of water and ultrasonicated for 60 min to uniformly disperse the carbon. Melamine, with various mass ratios (1:0.5; 1:1; 1:2.5; 1:5; and 1:10), with respect to the amount of carbon taken (carbon: melamine), was added into the carbon suspension, which was then ultrasonicated and then magnetically stirred overnight. Next, the add-mixture of carbon and melamine was then dried in a hot-air oven completely in order to produce dried power, which was then collected and ground into a fine powder using a motor and pestle. The fine powder was then transferred to a graphite boat and placed in the center of the tubular furnace and pyrolyzed at 900 °C for 1 h at the heating ramp rate of 5 °C/min, under a continuous flow of inert N_2_ gas. The resulting black powder collected from the graphite boat was then finely ground using a mortar and pestle. The finally obtained N-doped catalysts were designated as N-CC-1, 2, 3, 4, and 5, corresponding to the mass ratios (1:0.5; 1:1; 1:2.5; 1:5; and 1:10) of melamine with respect to the amount of carbon taken (carbon: melamine). We observed that the N doping performed to the raw-CC (without pyrolysis) exhibited enhanced ORR activity than the N-doping performed to the P-CC. Therefore, the N-CC-1, 2, 3, 4, and 5 catalysts correspond to the N-doped carbon synthetized from the raw-CC (without pyrolysis). The details of this will be discussed in the results and discussion section (Section 3).

### 2.3. Synthesis of Mn-Xerogel via Sol-Gel Synthesis and the MnO/N-CC Catalyst

Mn-Xerogel was synthesized via a sol-gel synthesis process by using potassium permanganate (KMNO_4_) as the Mn precursor and glucose as the reducing agent. Thus, 1.0 g of KMNO_4_ was dissolved in 30 mL of the distilled water and 4.78 g of glucose was dissolved in 30 mL of the distilled water in separate beakers. Then, the glucose solution was added to the KMNO_4_ solution; the mixture was quickly stirred with the glass rod. As a result, a dark black gel was formed after 2–3 min. After the gel formed, the supernatant water was removed periodically and then the gel-containing beaker was transferred into a hot-air oven to dry overnight at 60–70 °C slowly [28]. Then, the dried gel was ground into a fine powder and then pyrolyzed at the temperatures of 700, 800, and 900 °C (MnO/N-CC-2-700, 800, and 900 respectively) in a tubular furnace at the heating rate of 5 °C/min in an N_2_ atmosphere. The resulting powder after the pyrolysis was designated as MnO. For the synthesis of the MnO/N-doped carbon catalyst, the aforementioned process was repeated except that the glucose solution was also mixed with the N-CC carbons at different mass contents (50, 100, 200, 300, and 400 mg). Adding N-CC carbon did not have any significant change in the gelation time, where the xerogel formed within 2–3 min. The obtained gel was further processed in the gel preparation procedure discussed above. The MnO/N-CC catalysts obtained this way was designated as MnO/N-CC-2 900-1, 2, 3, 4, and 5.

All the N-CC and MnO/N-CC catalysts was subjected to various physicochemical characterizations such as XRD, BET, SEM, TEM, and XPS to observe the phase of the MnO graphitization of the N-CC carbons, to measure the surface area and pore size distribution, to understand the morphology of the N-CC and the MnO nanoparticles’ distribution on the N-CC support, and to assess the valance states of MnO and the various N-bonding configurations in the N-CC catalysts. To estimate the ORR kinetics, all the catalysts were systematically evaluated for ORR in a traditional three electrode system with the glassy carbon as the substrate, in a 0.1 M HClO_4_ solution via CV, LSV, and RDE techniques. The ORR activity of the N-CC and MnO/N-CC catalysts were finally compared with the Pt/C (10 wt.%) as the standard. In addition, the final MnO/N-CC catalyst was also utilized as a cathode catalyst in dual-chamber MFC. The MFC polarization and TOC analysis were performed to assess the ability of the MFC to degrade the organic pollutants from the wastewater. Detailed information on these procedures is given in the Supplementary Materials section of this article.

## 3. Results

**Figure 1:**
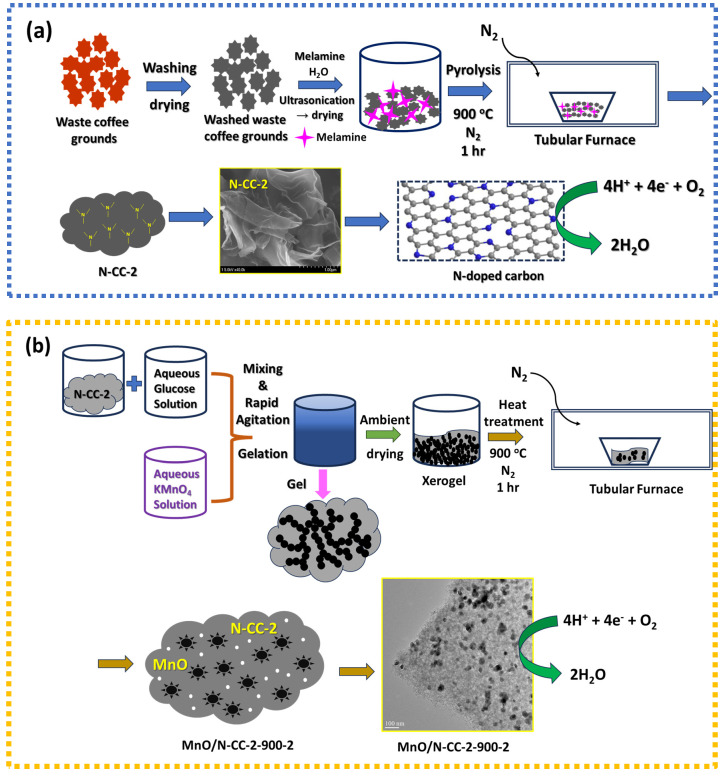
(**a**) Schematic of the synthesis of N-doped carbon from waste coffee ground. (**b**) Schematic of the synthesis of MnO/N-CC-2-900-2 catalyst via sol-gel synthesis with heat treatment.

The coffee waste obtained from the local wending machine is added to the ample of distilled water and magnetically stirred for 1 h, and then the solution is allowed to settle down. When the coffee waste was mixed with water and allowed to settle down, the supernatant water was initially found to be a dark brown color, which is believed to be the soluble organic compounds from the crushed and steamed coffee beans. This washing process is repeated several times until the supernatant water becomes clear, assuming that all the soluble impurities and organic compounds are eliminated. The resulting suspension is then dried and finely ground and used as a substrate for the N-doping. The N doping is performed with the melamine as the N precursor. When melamine is mixed with the waste coffee carbon, melamine is adsorbed on the carbon via π-π interactions and hydrogen bonding with the carbon functionalities [52]. When subjected to pyrolysis, the melamine undergoes decomposition into g-C_3_N_4_ at temperatures above 350 °C, and further increases in temperature leads to the formation of NH_3_ gas, which acts as a source of the N dopant. In parallel, the waste coffee carbon also undergoes graphitization at the higher temperatures and carbonizes into the carbon framework. During the graphitization, unstable oxygen functionalities undergo decomposition, which provides active sites for the doping of N into the carbon framework [53]. Therefore, during pyrolysis, carbon undergoes both N doping and graphitization at the same time. The optimum N content in the resultant carbon is adjusted by simply varying the melamine mass with respect to the carbon, and the pyrolysis process is repeated to obtain the N-doped carbon. The N-doped carbon with optimum ORR activity is established via RDE studies (as discussed in later sections). The optimized N-doped carbon is then used as a carbon support to deposit the MnO nanoparticles via a sol-gel synthesis route. In this study the manganese oxide is synthesized via the reduction of KMnO_4_ with organic reducing agents; in this case, the glucose sugar [27]. A sol-gel process involves the use of inorganic salts as precursors to achieve gelation via continuous hydrolysis and polycondensation processes [54]. The gel is formed within 2–3 min after adding the aqueous glucose solution to the aqueous KMnO_4_, which are stirred quickly together, and then the gel is left to stand. After occasionally pouring off water liberated during gel syneresis, a dark monolith gel is formed [55]. The resulting material is then dried at the temperature of 60–70 °C with slow heating until the gel completely dried. The resulting material is then used as an electrocatalyst for ORR (Figure 1).

**Figure 2:**
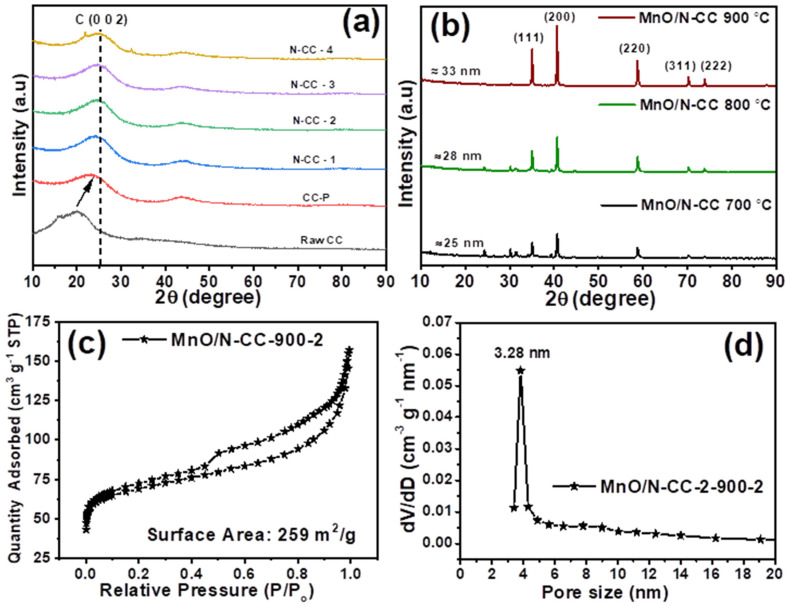
(**a**) XRD patterns to support the effect of heat treatment at 900 °C improving the graphitization and properties of N-doped carbons. (**b**) XRD patters to exhibit that MnO phase is formed in Mn-oxide and supported on N-CC catalysts at different temperatures, with increasing crystallization and size of MnO nanoparticles. (**c**) BET data to prove that the MnO/N-CC-900-2 catalyst exhibits a porous nature, similar to traditional Vulcan carbon support, enhancing oxygen reduction reaction (ORR) kinetics. (**d**) Pore size distribution data to support that the MnO/N-CC-900-2 catalyst contains predominant mesopores, facilitating efficient oxygen delivery and water removal, contributing to improved ORR kinetics.

Figure 2a shows the XRD patterns of the N-doped carbons synthesized from the different mass ratios of melamine:carbon. It is observed that the raw coffee carbon in this study contained an amorphous structure, as it can be seen from the lower 2 theta angles of the diffraction peaks associated with the graphitized carbons at ~26°, corresponding to C (002) plane. In contrast, the pyrolyzed carbon and N-doped carbons show better graphitization as the diffraction peaks shifts towards the 2 theta angles of ~26°, corresponding to C (002) plane. This indicates that the heat treatment at 900 °C helps to improve the graphitization of the carbon, which could expect better electronic conductivity and improved carbon corrosion resistance under electrochemical conditions. Figure 2b shows the XRD patterns of manganese oxide, supported on the N-CC catalyst, being heat treated at different temperatures. It is seen that all the catalysts showed typical diffraction patterns associated with the Mn in the form of MnO, as can be seen from how their diffraction patterns are positioned at 35.0, 40.5, 58.7, 70.0, and 73.9°, corresponding to the (1 1 1), (2 0 0), (2 2 0), (3 1 1), and (2 2 2) planes, respectively (PDF #01-075-6876). The formation of the MnO phase at temperatures higher than 800 °C is consistent with the literature reports [55,56]. It is well known that the phases of Mn_x_O_y_ are dependent on synthesis technique and the heat treatment temperatures (calcination temperature). When the Mn_x_O_y_ are heat treated at temperatures lower than 700 °C, the Mn_x_O_y_ exists in the form of MnO_2_/Mn_2_O_3_. However, with the increasing temperatures, a phase transformation is seen to occur from MnO_2_ → Mn_2_O_3_ → Mn_3_O_4_ → MnO. It is to be noted that MnO/MnO_2_/Mn_2_O_3_/Mn_3_O_4_, all of the forms of Mn_x_O_y_, are active towards the ORR. The similar type of temperature-dependent phase transformation of MnO_2_ → MnO has experimentally been observed and validated in several reports [56,57,58,59].

From Figure 2b, we can also observe that with the increasing heat treatment, the diffraction peaks’ intensity also increases, due to the increased crystallization degree and crystallite size of the MnO nanoparticles. The crystallite size of the MnO/N-CC catalysts are calculated considering the high intensity peak at 40.5° which corresponds to the (200) plane by using Scherrer equation. The MnO crystallite sizes of 25, 28, and 33 nm are obtained for the MnO/N-CC-700, 800, and 900 catalysts, respectively. From the detailed RDE studies (discussed in the later sections), it was found that the N-CC-2 and MnO/N-CC-2-900-2 catalyst showed best ORR activity; hence, the other physicochemical characterization such as BET, SEM, TEM, and XPS are only limited to the final optimized catalyst, i.e., MnO-N-CC-900. Figure 2c shows the N_2_ adsorption and desorption isotherms and the associated pore size distribution analysis using the BET method. The MnO/N-CC-900-2 catalyst showed a typical type IV isotherm with a hysteresis loop ranging from a relative pressure of 0.40 to 1.0 (P/P_o_), indicating a typical porous structure of the catalysts. Further, the surface area of 259 m^2^/g is obtained from the BET isotherm and this value is approximated to that of the traditional Vulcan carbon support used in the Pt/C catalyst [60]. Furthermore, the pore size distribution of the MnO/N-CC-900-2 catalyst is shown in Figure 2d, which appears to have predominant pores in the mesopore range around 3.28 nm, i.e., the mesopore range. The presence of mesopores is very essential to deliver the gaseous oxidant O_2_ to the MnO active sites and, at the same time, help in quickly removing the product water out from the catalytic active site, mitigating the mass transfer limitation issues, and therefore, contributing to the enhanced ORR kinetics [61].

**Figure 3:**
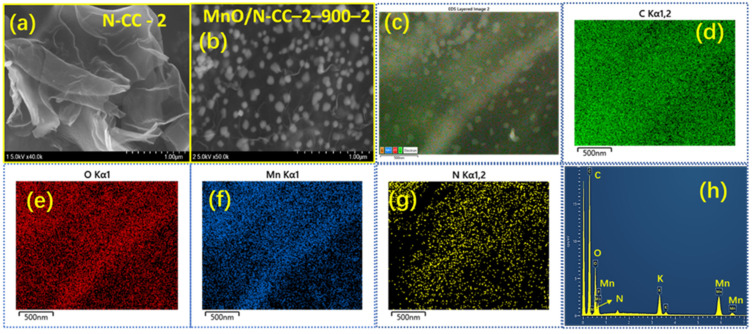
SEM images of (**a**) N-CC-2, (**b**) MnO/N-CC-2-900-2 catalysts, (**c**) SEM images of the EDS image used for the elemental mapping of (**d**) C, (**e**) O, (**f**) Mn, (**g**) N, and (**h**) EDAX spectrum of MnO/N-CC-2-900-2 catalysts.

Figure 3a,b shows the SEM images of the N-CC-2 and MnO/N-CC-2-900-2 catalysts. It is seen that the N-CC-2 catalyst showed as crumpled carbon sheets, whereas the MnO/N-CC-2-900-2 catalyst showed as the MnO nanoparticles supported on the N-CC-2 carbon sheets derived from the coffee waste. It is seen that MnO are well supported and covepotentialCC-2 carbon nanosheets, which guarantees the effective interfacial electron transfer during the ORR and also helps in mitigating the dissolution of the MnO nanoparticles in the acidic electrolytes under the potential cycling conditions, which therefore enhances the overall stability of the catalyst. The well-integrated MnO into the crumpled carbon sheets is possible due to the fact that the N-CC-2 is added to during the gel formation, as described in the experimental section (Section 2.2). This also ensures that the N-CC-2 carbon with sufficient surface area is available during the gel formation and the gel is directly formed on the N-CC-2 support, rather than adding the pre-formed MnO nanoparticles to the carbon sheets separately, as described in the study by Chander et al. [56].

**Figure 4:**
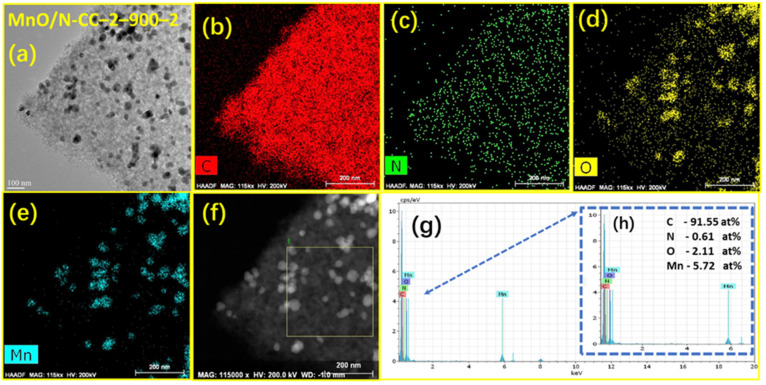
TEM image of the (**a**) MnO/N-CC-2-900-2 catalyst and the elemental mapping for (**b**) carbon, (**c**) N, (**d**) O, (**e**) Mn, and (**f**) images’ area used for EDAX spectra (**g**) obtained from EDAX spectra inset’s (**h**) zoomed portion of the EDAX spectrum with elemental composition in atomic.%.

The SEM images of the MnO nanoparticles synthesized via the sol-gel method in this work can be comparable to that of M. Minakshi et al. [62] for electrolytic manganese dioxide (EMD). The EMD grade MnO_2_ shows high crystallinity with large particle size, whereas the sol-gel-derived MnO nanoparticles in this study show similar crystallinity but with smaller particle size. Adding N-CC-2 during the gelation also helps in alleviating the agglomeration of the MnO nanoparticles during the drying of the gel, ensuring the nanoparticles are well separated from each other. In addition, the MnO/N-CC-2-900-2 catalysts’ elemental mapping and the energy-dispersive X-ray analysis (EDAX) analysis show the presence of C, N, O, and Mn in the catalyst (Figure 3c–h). To further understand the morphology of the catalyst in deep, HR-TEM, and high-angle annular dark-field (HAADF) imaging of the elements in the MnO/N-CC-2-900-2 catalysts is analyzed. Figure 4a shows the HR-TEM image of the MnO/N-CC-2-900-2 catalysts. It is clearly visible that the MnO nanoparticles are evenly distributed on the porous carbon support. No visible agglomeration of the nanoparticles was detected. The TEM morphology of the MnO/N-CC-2-900-2 catalysts are well supported via the SEM observations. Further, HAADF imaging clearly shows that the catalyst contains a thick sheet of carbon, nitrogen, oxygen, and the nanoparticles of manganese (Figure 4b–e). Further, the oxygen elemental mapping clearly distinguishes between the functionalized oxygen species on the carbon support and the oxygen atoms within the lattice of MnO. The oxygen atoms clustered consist of MnO nanoparticles (Figure 4e), whereas the scattered yellow colored oxygen atoms belong to the carbon support (Figure 4d). This observation clearly indicates that MnO nanoparticles are obtained from the xerogel obtained from the sol-gel synthesis process. Further, the identification and quantification of the elements in the MnO/N-CC-2-900-2 catalyst are obtained by recording the EDAX spectrum, which clearly shows the presence of carbon, nitrogen, oxygen, and manganese (Figure 4f–h).

**Figure 5:**
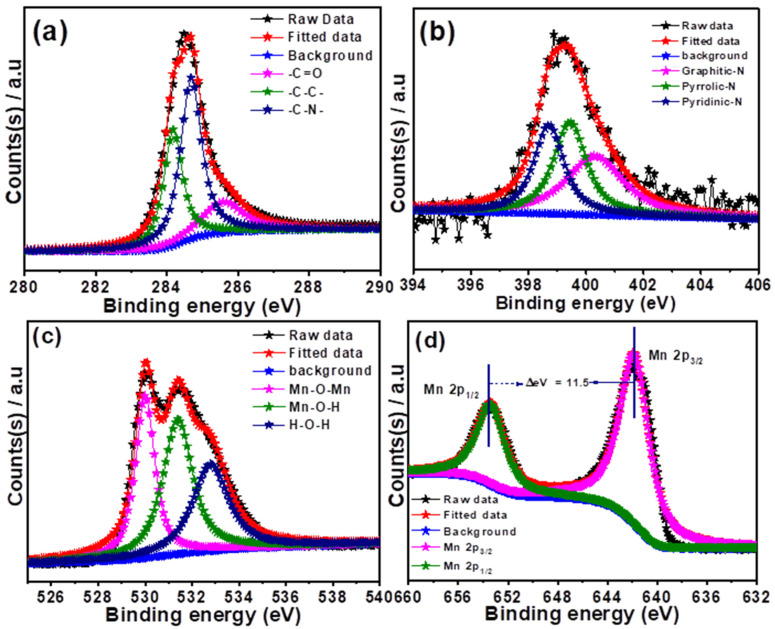
Deconvoluted XPS peaks for (**a**) C1s, (**b**) N1s, (**c**) O1s, and (**d**) Mn2p of MnO/N-CC-2-900-2 catalyst.

To obtain further insights into the chemical states of the elements present in the MnO/N-CC-2-900-2 catalyst, XPS analysis was carried out, and all the elements’ spectra were further deconvoluted. Figure S1 shows the XPS survey spectra of the MnO/N-CC-2-900-2 catalyst, which confirms the existence of the C, N, O, and Mn species. The C1s deconvoluted spectra (Figure 5a) show the peaks associated with the -C-C-, -C-N-, and -C=O- chemical bonding in the catalysts, which resulted from the hexagonal carbon framework, the N-doped into the carbon matrix, and the surface oxygen functionalities of the carbon at the binding energies of 284.2, 284.6, and 285.7 eV, respectively. The appearance of -C-N bonding clearly confirms that the N doping into the carbon matrix has been accomplished. Further, it is well known that the N doping into the carbon matrix results in a different bonding environment with carbon, namely, pyridinic-N, graphitic-N, and pyrrolic-N, which appears at different binding energy levels that could be identified from the high resolution deconvoluted spectra of N [31]. Figure 5b shows three different peaks at the binding energies of 398.6, 399.5, and 400.4 eV, corresponding to the pyridinic-N, pyrrolic-N, and graphitic-N species, respectively. It is well known that all of these forms are active for ORR, though there is ambiguity around which one of these bonding environments is truly active. However, there are enough evidence that the order of activity follows pyridinic-N > graphitic-N > pyrrolic-N. The doping of N is known to break the electroneutrality-induced electronic charge density and spin density of the carbon atoms, which serves as active catalytic centers for the ORR reaction [63,64]. In addition, N-doping helps in increasing the electronic conductivity of the catalysts and increasing the electronic density around the Mn catalytic active sites, resulting in enhanced ORR activity. Figure 5c shows the deconvoluted O1s spectra which shows three deconvoluted peaks at the binding energies of 529.9, 531.3, and 532.7 eV, corresponding to the O atom bonding in Mn-O-Mn, Mn-O-H, and C-OH [65,66]. Figure 5d shows the Mn2p spectra of the MnO/N-CC-2-900-2 catalyst, which showed a typical peak around 641.98 and 653.48 eV, which is attributed to the binding energy of the Mn 2p_3/2_ and Mn 2p_1/2_ states, respectively. The binding energy separation between c is found to be 11.50 eV, which is consistent with the literature reports on the MnO nanoparticles [67,68,69]. The XPS analysis prove the presence of C-N bonding, which implies the successful doping of N atoms into the carbon matrix. In addition, the existence of pyridinic-N, graphitic-N, and pyrrolic-N proves that the N-doped carbon support can perform the ORR both independently and together with assisting the MnO in enhancing the overall ORR activity by supplying electrons to the active sites. The existence of Mn-O-Mn and the typical charge separation between the binding energy of the Mn 2p_3/2_ and Mn 2p_1/2_ states clearly prove that the MnO nanoparticles are deposited on the N-doped carbon support; hence, any outstanding ORR activity can be expected to be shown in half-cell and the MFCs’ set up.

**Figure 6:**
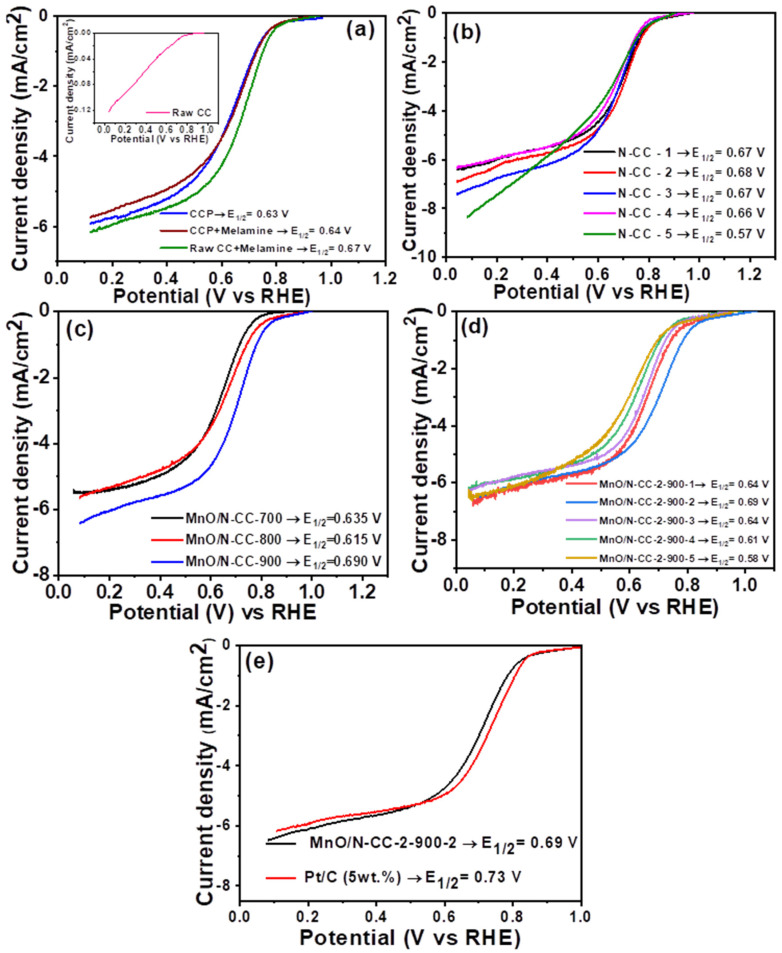
LSV curves of (**a**) CCP, CCP + melamine, raw-CC + melamine catalysts, (**b**) N-CC catalysts with different melamine contents, (**c**) MnO/N-CC-2 catalyst with heat treatment at different temperatures, (**d**) MnO/N-CC-2-900-2 catalysts synthesized with different contents of N-CC-2 carbon, and (**e**) LSV comparison of MnO/N-CC-2-900-2 catalysts with Pt/C (5 wt.%). All the LSV curves were recorded in O_2_ saturated with 0.1 M of HClO_4_ electrolyte, with a scan rate of 10 mV/s, and the room temperature condition.

To understand the ORR kinetics, all the N-CC and Mn/N-CC catalysts have been subjected to the RDE studies in 0.1 M of the HClO_4_ electrolyte. Traditionally, those (RDE studies) incorporate three electrode systems with a graphite rod as the counter electron, saturated calomel as the reference electrode, and the catalyst ink composed of the N-CC and Mn/N-CC catalysts are deposited on the glassy carbon electrode as the working electrode. All the experiments are conducted around 25 °C. First, N doping is provided onto raw CC (without pyrolysis) and after pyrolysis (CC-P), to understand the effect of surface defects and to conclude as to which of the carbons would be desirable to start the experiments with. Figure 6a shows the linear sweep voltametric (LSV) curves of the raw CC, CCP, raw-CC + melamine (raw-CC-N), and CCP + melamine (CCP-N) samples. It is clearly seen that the raw CC showed poor ORR characteristics (inset of Figure 6a), in contrast to the CCP, raw-CC-N, CCP-N catalysts which exhibited typical ORR characteristics with half-cell potentials of 0.63, 0.67, and 0.64 V respectively, confirming that CCP and N-doping to the raw CC (raw-CC-N) result in higher ORR activity than the CCP and CCP-N. It is quite obvious that trying to dope N into the previously pyrolyzed carbon would hardly result in the incorporation of nitrogen into the carbon matrix, given that during the pyrolysis, the carbon has already graphitized with fewer to no defects/oxygen-containing surface functional groups to assist in N doping. In contrast, raw-CC is found to have surface defects and an amorphous structure with insufficient oxygen-containing surface functional groups, which would provide sites for nitrogen doping, and hence, results in higher half-wave potentials. With this preliminary conclusion, all further experiments were conducted with raw-CC with different contents of melamine to understand the effect of the melamine precursor concentration on the ORR activity.

Figure 6b shows the linear sweep voltammetry curves recorded for the catalyst synthesized with different mass ratios of melamine relative to raw-CC. It is seen that all the catalysts showed typical ORR characteristics with a half-wave potential of 0.67, 0.68, 0.67, 0.66, and 0.57 V for the N-CC-1, 2, 3, 4, and 5 catalysts, respectively. It is seen that, with the increasing melamine concentration, the half-wave potentials gradually increased from 0.67 to 0.68 V for the N-CC-1, 2 catalysts, due to the higher proportion of nitrogen species generated from the melamine decomposition, generation of ammonia, and incorporation of N into the carbon matrix. However, the further increase in the mass ratio of melamine resulted in the decreased half-wave potential attributing to the decrease in ORR activity, indicating that the optimum ratio of nitrogen doping has surpassed despite the possible high percentage of nitrogen doping. Among all the catalysts, N-CC-2, corresponding to the raw-CC:melamine ratio of (1:1), was found to have a maximum half- wave potential of 0.68 V; therefore, all the subsequent experiments were conducted with the N-CC-2 catalyst. Figure 6c shows the LSVs for the MnO nanoparticles deposited on the N-CC2 support that undergoes the heat treatment at 700, 800, and 900 °C. Among those, the MnO/NCC-2 900 °C catalyst showed the best ORR activity with a half-wave potential of 0.690 V. The higher ORR activity of the MnO/NCC-2 900 catalyst could be attributed to higher crystallinity and the even distribution of the MnO nanoparticles on the nitrogen-doped carbon support. Further, to understand the optimum nitrogen-doped carbon support needed for the best ORR activity, we proportionately increased the N-CC carbon support that was added during the generation of the manganese oxide xerogel. Figure 6d shows the ORR polarization curves for various catalysts with increasing N-CC content. Among all the catalysts, the catalyst synthesized with 100 mg of the N-CC MnO/N-CC-2-900-2 showed better ORR half-wave potentials of 0.69 V. Finally, the optimized MnO/N-CC-2-900-2 catalyst ORR activity is compared with the commercial Pt/C catalyst (5 wt.%), as shown in Figure 6e. The Pt/C catalyst showed a half-wave potential of 0.73 V, whereas the MnO/N-CC-2-900-2 catalyst was 0.69 V. The MnO/N-CC-2-900-2 catalyst is still interior to the Pt/C catalyst by 40 mV. Although the MnO/N-CC-2-900-2 catalysts with improved ORR activity did not meet the commercial standard, they are still promising because the Mn-based catalysts are less expensive than the noble metal Pt-based catalysts.

**Figure 7:**
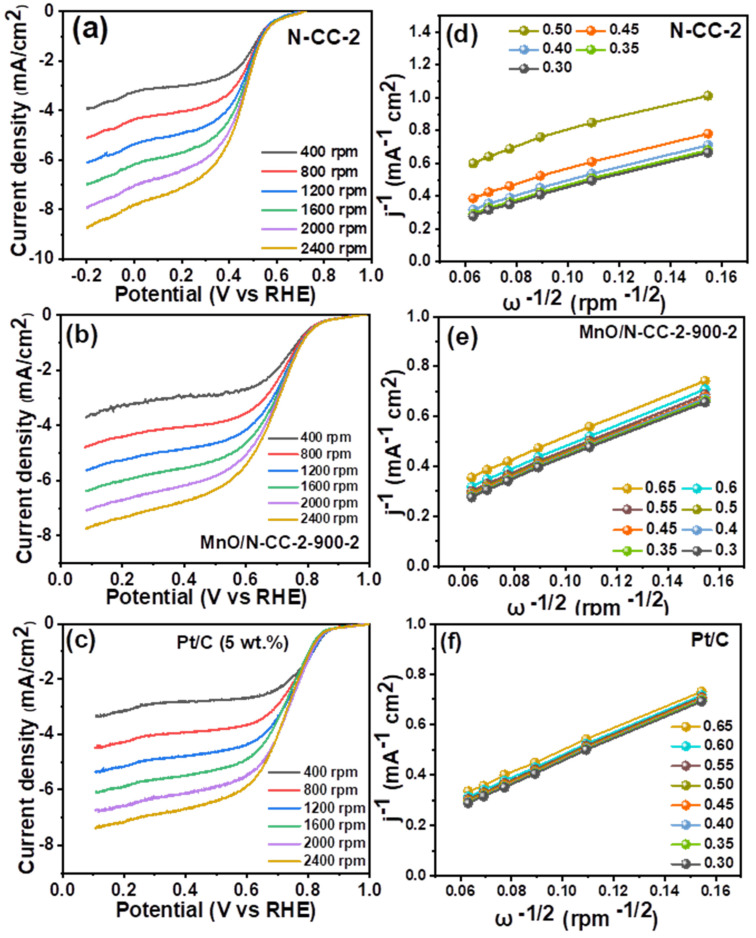
LSV curves recorded at different rotations per minute and their respective K-L plots.

Taking readings of the LSV of the N-CC-2, MnO/N-CC-2-900-2, and Pt/C catalysts at various rotations (Figure 7a–c) and then constructing the K-L plots (Figure 7d–f) provide insight on the ORR pathway. It is well known that ORR occurs in either a direct 4-electron reduction or a 2 + 2 electron reduction [31]. A direct 4-electron reduction pathway results in the formation of H_2_O as a final product, whereas a 2 + 2 reduction pathway results in the formation of undesirable H_2_O_2_, which further undergoes decomposition and reduction to H_2_O. The former pathway leads to higher efficiency, whereas the latter one leads to less efficiency of the ORR process. Moreover, the formation of H_2_O_2_ causes the degradation of the catalyst support and the membrane; hence, a direct 4-electron pathway or near to the 4-electron pathway is essential for the ORR catalyst. In order to estimate the ORR process’ efficiency, the K-L plots (i.e., the plots of inverse of the square root of rotation speed versus the inverse of the current density) are utilized to determine the number of electrons involved in the ORR process. Figure 7a–c exhibits the ORR curves recorded at different rotations which show that ORR is O_2_ diffusion-controlled reactions, with the limiting current increasing as the rpm for all the three catalysts increases, without any noticeable change at the onset potential. This indicates that the ORR kinetics are similar at all rpms and the only change observed in the mixed kinetics-diffusion controlled and diffusion-controlled regions of the LSV curves. In addition, the linear K-L plots (Figure 7d–f) for all the three catalysts also suggest that an almost similar electron transfer number can be predicted at all the potentials. The calculated number of electrons were found to be an average of 3.91, 3.94, and 3.98 electrons for the N-CC-2, MnO/N-CC-2-900-2 and Pt/C catalysts. Although some 2 + 2 electron reduction is still possible, the obtained ‘n’ close to 4 indicates that the catalyst primarily uses a direct 4-electron pathway to perform ORR.

In addition to the ORR activity, the stability of the catalyst is one of the major concerns in the cathode of MFC and other energy conversion devices such as fuel cells and metal–air batteries. It is well known that the traditional Pt/C catalyst undergoes degradation by several mechanisms such as carbon corrosion, Pt nanoparticle dissolution, agglomeration and sintering, and the Ostwald ripening phenomenon [31]. This results from the fact that the traditional Pt/C catalyst uses Vulcan carbon as a support which is known to have less graphitization and sensitivity to carbon conversion. To understand the stability of the Pt/C and MnO/N-CC-2-900-2 catalyst, we performed a repeated potential cycling test via the repetitive recording of cyclic voltammetry curves in O_2_ saturated with the 0.1 M HClO_4_ electrolyte. It is well known that the potential cycling test promotes carbon corrosion and metal dissolution during the oxidative and reductive scan by using cyclic voltammetry. This repetitive cycling test is performed for 20,000 potential cycles with LSVs recorded at specific intervals and the stability of the catalyst wear is assessed by calculating loss in the half-wave potentials.

**Figure 8:**
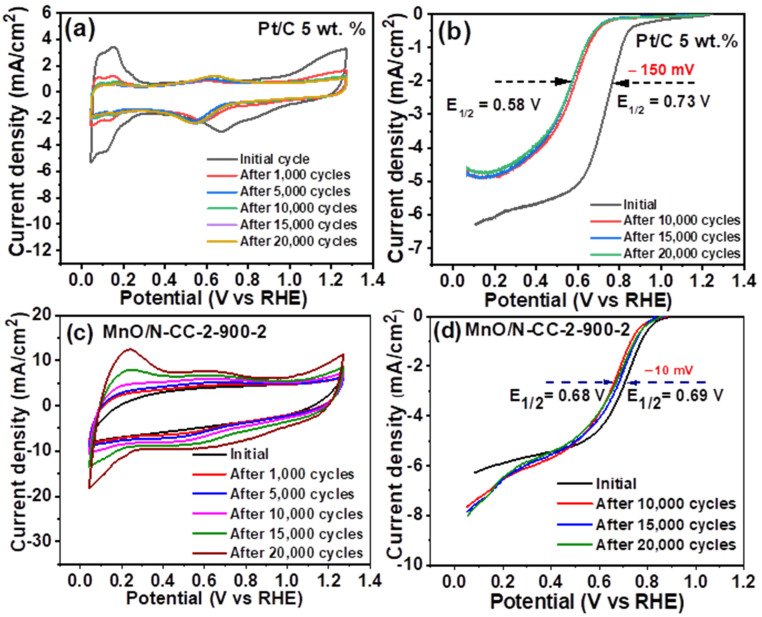
Potential cycling stability test results. Cyclic voltammetry curves recorded before and after repeated potential cycles of (**a**) Pt/C catalyst, (**b**) MnO/N-CC-2-900-2 catalyst and their LSV curves, (**c**) Pt/C, and (**d**) MnO/N-CC-2-900-2 catalyst, respectively.

Figure 8a shows the CV curves of the Pt/C catalyst in the 0.1 M HClO_4_ electrolyte. The CV curve show typical H_2_ adsorption/desorption characteristics between the potentials of 0–0.4 V and Pt-oxide and Pt-OH at the potentials between 0.6–1.2 V. In general, the integrated area under the H_2_ adsorption/desorption region represents the electrochemical surface area (ECSA) of the platinum nanoparticles available for ORR. Higher ECSA leads to a higher surface area of platinum available for ORR. It is well known that under potential cycling conditions, the carbon corrosion, platinum dissolution, redeposition, and agglomerations speeds up, and those lead to a decreased area under the H_2_ adsorption/desorption region, in turn leading to a loss of ECSA and ORR activity. In line with this, one can observe the drastic reduction in the H_2_ adsorption/desorption regions in the CV curves. In addition, the peak potential for Pt-OH are also shifted towards the lower potentials after cycling the catalyst for 20,000 potential cycles. Figure 8b shows the LSV curves for the Pt/C catalyst recorded after 10, 15, and 20,000 potential cycles. It is clearly seen that the Pt/C catalyst’s onset and half-wave potentials shifts negatively with increasing potential cycles. The half-wave potential shifts from 0.73 V to 0.58 V, with an overall loss of 150 mV after 20,000 potential cycles. This is because of the loss of ECSA resulting from the carbon corrosion, Pt nanoparticle dissolution, re-deposition, and agglomeration, as can be seen from the CV curves. This result indicates that, despite initial better ORR kinetics, the Pt/C catalyst lose its electrocatalytic ORR kinetics gradually with potential cycles, which proves its poor stability over longer periods of time. In contrast, the CV curves of the MnO/N-CC-2-900-2 catalyst did not show any signs of loss in ECSA; in fact, the capacitance region was still enhanced after potential cycling, which could be attributed to the increased exposure of the MnO nanoparticles due to the porous carbon support (Figure 8c). The LSV curves recorded after 10, 15, and 20,000 potential cycles indicate that the onset and half-wave potentials were nearly unchanged after 20,000 potential cycles, where a calculated loss of just 10 mV of the half-wave potential is observed (Figure 8d). This clearly indicates that the MnO/N-CC-2-900-2 catalyst is highly stable under potential conditions, which is far better than the commercial Pt/C standards. The better stability of the MnO/N-CC-2-900-2 catalyst over traditional Vulcan carbon in the Pt/C catalyst could be attributed to several factors: (i) the increased corrosion resistance of N-doped carbon, (ii) the enhanced electronic conductivity of the N-doped carbon due to the presence of pyridinic-N and graphitic-N species, (iii) the intrinsic ORR ability of N-doped carbon which has the ability to perform ORR independently and also assists in the ORR kinetics of the supported MnO nanoparticles, (iv) the strong metal–support interaction between the N-doped carbon support and the MnO nanoparticles, exclusively due to the N doping’s (v) high crystallinity of the MnO nanoparticles, which mitigates the dissolution during the potential cycling conditions [31]. In short, the stability test clearly indicates that when MnO/N-CC-2-900-2 is used as the ORR catalyst in MFCs, or in fact, in any other ORR bearing electrochemical energy conversion devices like fuel cells and metal–air batteries, the cathodic ORR kinetics could be well retained over a longer period than when the Pt/C catalyst is used.

After evaluating the MnO/N-CC-2-900-2 catalyst via various physical chemical characterizations and electrochemical characterizations, we finally tested this catalyst as cathodes and evaluated its performance in the dual-chamber MFC’s (DCMFC) set up. The catalyst ink made of the MnO/N-CC-2-900-2 catalyst is brush coated on the commercial gas diffusion layers with a catalyst loading of approximately 2 mg cm^−2^. The MFC performance was analyzed in a previously activated DCMFC cell operated with the brush anodes and inoculated with sewage water sludge collected from the local drainage as a source of bacteria and a mixture of sodium acetate and glucose as the carbon source. After introducing the cathode catalyst into the cathode compartment containing a phosphate buffer solution (PBS), the buffer continuously purged with atmospheric air, OCV was generated and continuously increased over a period of time, and the i–v curves were recorded only after attaining a stable voltage. For the comparison, a Pt/C-coated gas diffusion layer was also used in a separate experiment to evaluate the MFC performance.

**Figure 9:**
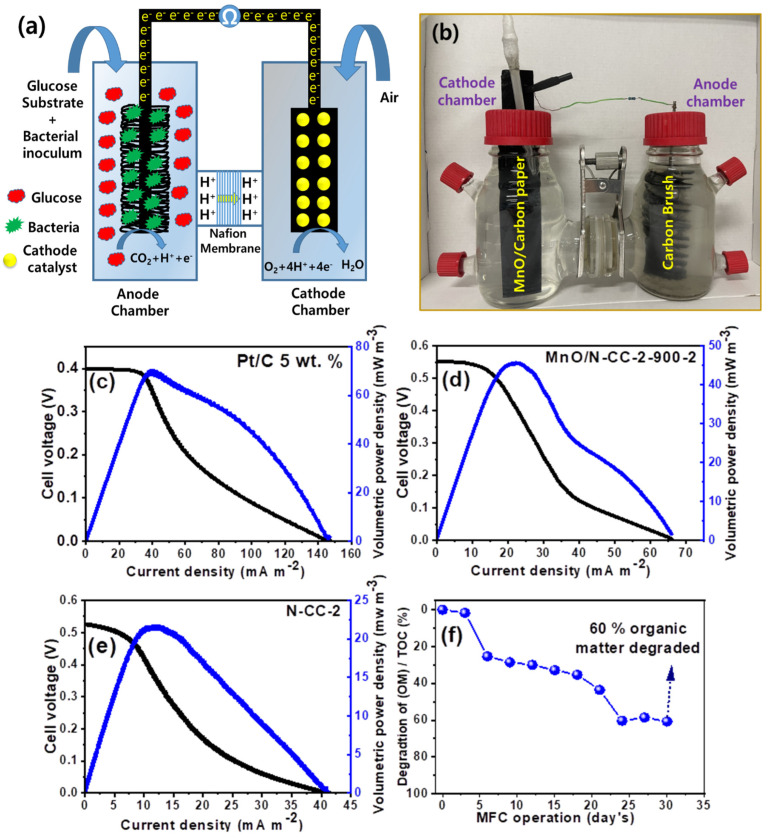
(**a**) Pictorial representation of dual-chamber microbial fuel cells and (**b**) laboratory working set-up of dual-chamber microbial fuel cells. i–v curves of (**c**) Pt/C and (**d**) MnO/N-CC-2-900-2 catalyst. (**e**) N-CC (**f**) TOC results of the anode chamber of the dual-chamber microbial fuel cells with MnO/N-CC-2-900-2 catalyst.

Figure 9a shows the i–v curve for the Pt/C catalyst which exhibited the OCV of ~0.4 V and a volumetric power density of ~70 mW m^−3^. Figure 9d,e shows the i–v curves of the MnO/N-CC-2-900-2 and N-CC-2 catalyst which exhibited the OCV higher than the Pt/C catalyst, i.e., ~0.55 V and 150 mV higher than the Pt/C catalyst. This further confirms the better ORR kinetics on the MnO/N-CC-2-900-2 catalyst than on the Pt/C catalyst. The volumetric power density was measured at ~45 mW m^−3^, whereas N-CC-5-2 delivered 21 mW m^−3^. The higher volumetric power density is attributed to the MnO nanoparticles supported on the N-doped carbon derived from coffee waste. The MFC performance obtained in this study is rather low. We believe that high catalyst loading (4 mg cm^−2^) might be one of the reasons for the lower performance. High catalyst loading might decrease the oxygen diffusion rate from the bulk solution to the catalyst surface, in addition to several other reasons on the catalyst ink formulation, such as the Nafion content. Therefore, further improvements in the power density is possible with the optimization of the cathode catalyst loading and Nafion content, improving the porosity (by introducing pore-forming agents) into the catalyst layer, which would result in improved mass transfer-related issues and also changes the cathode backing layer from carbon paper to carbon felt, carbon foam, carbon brush, etc., which would be considered as a continuation of the present work in the future. In addition to these factors, the electrode surface area to anode volume ratio also significantly affects the MFC performance. We have a future plan to improve the MFC performance by changing the catalyst loading, optimizing the Nafion content, changing the anode and cathode substrate types (carbon brush, carbon felt, carbon mesh, stainless steel mesh, etc.), and the electrode surface area to anode volume ratio, aiming to improve the MFC performance and power output.

In addition to the ORR performance on the cathodes, we also evaluated the MFCs’ ability to degrade the organic pollutants in the anode chamber. In general, the measurement of the chemical oxygen demand (COD) is applied traditionally to assess the ability of MFC to degrade organic pollutants [70]. COD is a traditional water quality measurement technique which is also subjected to various errors. Instead, in the present study, we used the total organic carbon (TOC) measurement to understand the progress of organic contaminant degradation [71]. The MFC is operated with a 100 Ω resister and an ample of solution from the anode chamber is collected at specified intervals and analyzed for TOC measurement. Figure 9c shows the TOC values obtained, which show that a gradual decrease in the TOC values is identified, attributing to the oxidation of organic matter by the bacteria, and after continuous operation of MFC for 30 days, it is observed that 60% of the organic matter is degraded. This indicates that the MFC developed in this study is effective in degrading organic matter and, at the same time, generating an ample amount of power density.

## 4. Conclusions

In this study, we developed the MnO-based ORR catalysts via a one-step sol-gel synthesis process. The MnO nanoparticles were supported on the carbon support derived from the cheap sustainable source, i.e., waste coffee carbon. The nitrogen-doped carbon derived from the waste coffee powder was found to have a sheet-like morphology and the porosity suitable for the carbon support characteristics. The sol-gel-derived spherical MnO nanoparticles supported on the N-doped carbon were found to have a particle size of 33 nm and good crystallinity. The formation of the MnO nanoparticles was clearly identified from the XRD, TEM, and XPS analyses. The systematic investigation on ORR activity accessed in the acidic electrolytes shows that the MnO/N-CC-2-900-2 catalyst exhibited a similar ORR kinetics half-wave potential of 0.69 V and a nearly dominant, direct effect for the electron reduction process. In addition to the enhanced ORR activity, the MnO/N-CC-2-900-2 catalyst also exhibited excellent stability with just a loss of 10 mV in the half-wave potential after 20,000 potential cycles, which is much better than the traditional Pt/C catalyst which lost about 150 mV after 20,000 potential cycles. In DCMFC, the MnO/N-CC-2-900-2 catalyst exhibited higher open circuit voltage potentials close to 0.55 V when compared to 0.40 V for the Pt/C catalyst, with a volumetric power density of ~45 mW m^2^. Further, the TOC analysis revealed that the MFC constructed in this study could degrade 60% of the organic matter in a continuous operation of MFC for 30 days. All the above claims confirm that the developed MnO/N-CC-2-900-2 catalyst could be one of the cheapest alternatives for the MFC cathodes.

## Data Availability

Data are contained within the article.

## Supplementary Materials

The following supporting information can be downloaded at: https://www.mdpi.com/article/10.3390/nano13222949/s1, The RDE experimental procedures, the dual-chamber microbial fuel cell set up and procedures, and the XPS survey spectra are given in the supporting information.

## References

[1] Vishwanathan A.S. (2021). Microbial Fuel Cells: A Comprehensive Review for Beginners. 3 Biotech.

[2] Boas J.V., Oliveira V.B., Simões M., Pinto A.M.F.R. (2022). Review on Microbial Fuel Cells Applications, Developments and Costs. J. Environ. Manag..

[3] Aghababaie M., Farhadian M., Jeihanipour A., Biria D. (2015). Effective Factors on the Performance of Microbial Fuel Cells in Wastewater Treatment—A Review. Environ. Technol. Rev..

[4] Li S., Ho S.-H., Hua T., Zhou Q., Li F., Tang J. (2021). Sustainable Biochar as an Electrocatalysts for the Oxygen Reduction Reaction in Microbial Fuel Cells. Green Energy Environ..

[5] Peera S.G., Maiyalagan T., Liu C., Ashmath S., Lee T.G., Jiang Z., Mao S. (2021). A Review on Carbon and Non-Precious Metal Based Cathode Catalysts in Microbial Fuel Cells. Int. J. Hydrogen Energy.

[6] Ma J., Zhang J., Zhang Y., Guo Q., Hu T., Xiao H., Lu W., Jia J. (2023). Progress on Anodic Modification Materials and Future Development Directions in Microbial Fuel Cells. J. Power Sources.

[7] Malik S., Kishore S., Dhasmana A., Kumari P., Mitra T., Chaudhary V., Kumari R., Bora J., Ranjan A., Minkina T. (2023). A Perspective Review on Microbial Fuel Cells in Treatment and Product Recovery from Wastewater. Water.

[8] Kumar S.D., Yasasve M., Karthigadevi G., Aashabharathi M., Subbaiya R., Karmegam N., Govarthanan M. (2022). Efficiency of Microbial Fuel Cells in the Treatment and Energy Recovery from Food Wastes: Trends and Applications—A Review. Chemosphere.

[9] Jadhav D.A., Das I., Ghangrekar M.M., Pant D. (2020). Moving towards Practical Applications of Microbial Fuel Cells for Sanitation and Resource Recovery. J. Water Process. Eng..

[10] Ashmath S., Kwon H.-J., Peera S.G., Lee T.G. (2022). Solid-State Synthesis of Cobalt/NCS Electrocatalyst for Oxygen Reduction Reaction in Dual Chamber Microbial Fuel Cells. Nanomaterials.

[11] Che Z., Yuan Y., Qin J., Li P., Chen Y., Wu Y., Ding M., Zhang F., Cui M., Guo Y. (2023). Progress of Nonmetallic Electrocatalysts for Oxygen Reduction Reactions. Nanomaterials.

[12] Kiani M., Tian X.Q., Zhang W. (2021). Non-Precious Metal Electrocatalysts Design for Oxygen Reduction Reaction in Polymer Electrolyte Membrane Fuel Cells: Recent Advances, Challenges and Future Perspectives. Coord. Chem. Rev..

[13] Wang Y., Hao J., Liu Y., Liu M., Sheng K., Wang Y., Yang J., Li J., Li W. (2023). Recent Advances in Regulating the Performance of Acid Oxygen Reduction Reaction on Carbon-Supported Non-Precious Metal Single Atom Catalysts. J. Energy Chem..

[14] Shen H., Thomas T., Rasaki S.A., Saad A., Hu C., Wang J., Yang M. (2019). Oxygen Reduction Reactions of Fe-N-C Catalysts: Current Status and the Way Forward. Electrochem. Energy Rev..

[15] Shen S., Sun Y., Sun H., Pang Y., Xia S., Chen T., Zheng S., Yuan T. (2022). Research Progress in ZIF-8 Derived Single Atomic Catalysts for Oxygen Reduction Reaction. Catalysts.

[16] Türk K.K., Kruusenberg I., Kibena-Põldsepp E., Bhowmick G.D., Kook M., Tammeveski K., Matisen L., Merisalu M., Sammelselg V., Ghangrekar M.M. (2018). Novel Multi Walled Carbon Nanotube Based Nitrogen Impregnated Co and Fe Cathode Catalysts for Improved Microbial Fuel Cell Performance. Int. J. Hydrogen Energy.

[17] Bhowmick G.D., Kibena-Põldsepp E., Matisen L., Merisalu M., Kook M., Käärik M., Leis J., Sammelselg V., Ghangrekar M.M., Tammeveski K. (2019). Multi-Walled Carbon Nanotube and Carbide-Derived Carbon Supported Metal Phthalocyanines as Cathode Catalysts for Microbial Fuel Cell Applications. Sustain. Energy Fuels.

[18] Dhanabalan K., Perumalsamy M., Sriram G., Murugan N., Shalu, Sadhasivam T., Oh T.H. (2023). Metal–Organic Framework (MOF)-Derived Catalyst for Oxygen Reduction Reaction (ORR) Applications in Fuel Cell Systems: A Review of Current Advancements and Perspectives. Energies.

[19] Yang C., Ma X., Zhou J., Zhao Y., Xiang X., Shang H., Zhang B. (2022). Recent Advances in Metal-Organic Frameworks-Derived Carbon-Based Electrocatalysts for the Oxygen Reduction Reaction. Int. J. Hydrogen Energy.

[20] Ren J., Dyosiba X., Musyoka N.M., Langmi H.W., Mathe M., Liao S. (2017). Review on the Current Practices and Efforts towards Pilot-Scale Production of Metal-Organic Frameworks (MOFs). Coord. Chem. Rev..

[21] Barkholtz H.M., Liu D.-J. (2017). Advancements in Rationally Designed PGM-Free Fuel Cell Catalysts Derived from Metal–Organic Frameworks. Mater. Horizons.

[22] Peera S.G., Liu C., Asokan A., Suss M.E. (2023). Cu@NC as High-Performance and Durable Electrocatalyst for Oxygen Reduction Reaction in Alkaline Membrane Fuel Cells. J. Alloys Compd..

[23] Sgarbi R., Kumar K., Jaouen F., Zitolo A., Ticianelli E.A., Maillard F. (2021). Oxygen Reduction Reaction Mechanism and Kinetics on M-NxCy and M@N-C Active Sites Present in Model M-N-C Catalysts under Alkaline and Acidic Conditions. J. Solid State Electrochem..

[24] Dessie Y., Tadesse S., Eswaramoorthy R., Abebe B. (2019). Recent Developments in Manganese Oxide Based Nanomaterials with Oxygen Reduction Reaction Functionalities for Energy Conversion and Storage Applications: A Review. J. Sci. Adv. Mater. Devices.

[25] Wickramaarachchi K., Minakshi M. (2022). Status on Electrodeposited Manganese Dioxide and Biowaste Carbon for Hybrid Capacitors: The Case of High-Quality Oxide Composites, Mechanisms, and Prospects. J. Energy Storage.

[26] Biswal A., Tripathy B.C., Subbaiah T., Meyrick D., Minakshi M. (2013). Electrodeposition of Manganese Dioxide: Effect of Quaternary Amines. J. Solid State Electrochem..

[27] Brock S.L., Duan N., Tian Z.R., Giraldo O., Zhou H., Suib S.L. (1998). A Review of Porous Manganese Oxide Materials. Chem. Mater..

[28] Reddy R.N., Reddy R.G. (2003). Sol–Gel MnO_2_ as an Electrode Material for Electrochemical Capacitors. J. Power Sources.

[29] Sun Q., Li X.-H., Wang K.-X., Ye T.-N., Chen J.-S. (2023). Inorganic Non-Carbon Supported Pt Catalysts and Synergetic Effects for Oxygen Reduction Reaction. Energy Environ. Sci..

[30] Peera S.G., Koutavarapu R., Akula S., Asokan A., Moni P., Selvaraj M., Balamurugan J., Kim S.O., Liu C., Sahu A.K. (2021). Carbon Nanofibers as Potential Catalyst Support for Fuel Cell Cathodes: A Review. Energy Fuels.

[31] Zhang W., Chang J., Yang Y. (2023). Strong Precious Metal–Metal Oxide Interaction for Oxygen Reduction Reaction: A Strategy for Efficient Catalyst Design. SusMat.

[32] Zhou Y., Neyerlin K., Olson T.S., Pylypenko S., Bult J., Dinh H.N., Gennett T., Shao Z., O’Hayre R. (2010). Enhancement of Pt and Pt-Alloy Fuel Cell Catalyst Activity and Durability via Nitrogen-Modified Carbon Supports. Energy Environ. Sci..

[33] Lv Q., Si W., He J., Sun L., Zhang C., Wang N., Yang Z., Li X., Wang X., Deng W. (2018). Selectively Nitrogen-Doped Carbon Materials as Superior Metal-Free Catalysts for Oxygen Reduction. Nat. Commun..

[34] Hornberger E., Merzdorf T., Schmies H., Hübner J., Klingenhof M., Gernert U., Kroschel M., Anke B., Lerch M., Schmidt J. (2022). Impact of Carbon N-Doping and Pyridinic-N Content on the Fuel Cell Performance and Durability of Carbon-Supported Pt Nanoparticle Catalysts. ACS Appl. Mater. Interfaces.

[35] Goswami A.D., Trivedi D.H., Jadhav N.L., Pinjari D.V. (2021). Sustainable and Green Synthesis of Carbon Nanomaterials: A Review. J. Environ. Chem. Eng..

[36] Szabó A., Perri C., Csató A., Giordano G., Vuono D., Nagy J.B. (2010). Synthesis Methods of Carbon Nanotubes and Related Materials. Materials.

[37] Erb K.-H., Gingrich S. (2022). Biomass—Critical Limits to a Vital Resource. One Earth.

[38] Cavicchioli R., Ripple W.J., Timmis K.N., Azam F., Bakken L.R., Baylis M., Behrenfeld M.J., Boetius A., Boyd P.W., Classen A.T. (2019). Scientists’ Warning to Humanity: Microorganisms and Climate Change. Nat. Rev. Microbiol..

[39] Zhang Y., Pan H., Zhou Q., Liu K., Ma W., Fan S. (2023). Biomass-Derived Carbon for Supercapacitors Electrodes—A Review of Recent Advances. Inorg. Chem. Commun..

[40] Liang K., Chen Y., Wang D., Wang W., Jia S., Mitsuzakic N., Chen Z. (2023). Post-Modified Biomass Derived Carbon Materials for Energy Storage Supercapacitors: A Review. Sustain. Energy Fuels.

[41] Das S., Ghosh S., Kuila T., Murmu N.C., Kundu A. (2022). Biomass-Derived Advanced Carbon-Based Electrocatalysts for Oxygen Reduction Reaction. Biomass.

[42] Cao Y., Sun Y., Zheng R., Wang Q., Li X., Wei H., Wang L., Li Z., Wang F., Han N. (2023). Biomass-Derived Carbon Material as Efficient Electrocatalysts for the Oxygen Reduction Reaction. Biomass Bioenergy.

[43] Mehmandoust M., Li G., Erk N. (2023). Biomass-Derived Carbon Materials as an Emerging Platform for Advanced Electrochemical Sensors: Recent Advances and Future Perspectives. Ind. Eng. Chem. Res..

[44] Pagett M., Teng K.S., Sullivan G., Zhang W. (2023). Reusing Waste Coffee Grounds as Electrode Materials: Recent Advances and Future Opportunities. Glob. Chall..

[45] Akula S., Sahu A.K. (2019). Heteroatoms Co-Doping (N, F) to the Porous Carbon Derived from Spent Coffee Grounds as an Effective Catalyst for Oxygen Reduction Reaction in Polymer Electrolyte Fuel Cells. J. Electrochem. Soc..

[46] Srinu A., Peera S.G., Parthiban V., Bhuvaneshwari B., Sahu A.K. (2018). Heteroatom Engineering and Co-Doping of N and P to Porous Carbon Derived from Spent Coffee Grounds as an Efficient Electrocatalyst for Oxygen Reduction Reactions in Alkaline Medium. ChemistrySelect.

[47] Ramasahayam S.K., Azam S., Viswanathan T. (2015). Phosphorous, Nitrogen Co-Doped Carbon from Spent Coffee Grounds for Fuel Cell Applications. J. Appl. Polym. Sci..

[48] Kim B.G., Lee S., Jung Y.K., Lee J.U., Shin S., Shin T.H., Choi S.-M. (2023). Highly Durable Platinum Catalysts on Nano-SiC Supports with an Epitaxial Graphene Nanosheet Layer Grown from Coffee Grounds for Proton Exchange Membrane Fuel Cells. ACS Appl. Energy Mater..

[49] Li G., Sha J., Sun L., Jin R., Fu T., Xiang Y., Tang Y., Lei Y., Si Y., Guo C. (2022). Biomass Coffee Grounds Derived Nitrogen-Doped Ultrafine Carbon Nanoparticles as an Efficient Electrocatalyst to Oxygen Reduction Reaction. J. Alloys Compd..

[50] Ghouri Z.K., Al-Meer S., Barakat N.A.M., Kim H.Y. (2017). ZnO@C (Core@shell) Microspheres Derived from Spent Coffee Grounds as Applicable Non-Precious Electrode Material for DMFCs. Sci. Rep..

[51] Chung D.Y., Son Y.J., Yoo J.M., Kang J.S., Ahn C.-Y., Park S., Sung Y.-E. (2017). Coffee Waste-Derived Hierarchical Porous Carbon as a Highly Active and Durable Electrocatalyst for Electrochemical Energy Applications. ACS Appl. Mater. Interfaces.

[52] Liang Q., Shao B., Tong S., Liu Z., Tang L., Liu Y., Cheng M., He Q., Wu T., Pan Y. (2021). Recent Advances of Melamine Self-Assembled Graphitic Carbon Nitride-Based Materials: Design, Synthesis and Application in Energy and Environment. Chem. Eng. J..

[53] Peera S.G., Sahu A.K., Arunchander A., Bhat S.D., Karthikeyan J., Murugan P. (2015). Nitrogen and Fluorine Co-Doped Graphite Nanofibers as High Durable Oxygen Reduction Catalyst in Acidic Media for Polymer Electrolyte Fuel Cells. Carbon N. Y..

[54] Song H., Xu L., Chen M., Cui Y., Wu C., Qiu J., Xu L., Cheng G., Hu X. (2021). Recent Progresses in the Synthesis of MnO 2 Nanowire and Its Application in Environmental Catalysis. RSC Adv..

[55] Sarkar I.J.R., Peera S.G., Chetty R. (2018). Manganese Oxide Nanoparticles Supported Nitrogen-Doped Graphene: A Durable Alkaline Oxygen Reduction Electrocatalyst. J. Appl. Electrochem..

[56] Arunchander A., Vivekanantha M., Peera S.G., Sahu A.K. (2016). MnO–Nitrogen Doped Graphene as a Durable Non-Precious Hybrid Catalyst for the Oxygen Reduction Reaction in Anion Exchange Membrane Fuel Cells. RSC Adv..

[57] Gil A., Gandía L.M., Korili S.A. (2004). Effect of the Temperature of Calcination on the Catalytic Performance of Manganese- and Samarium-Manganese-Based Oxides in the Complete Oxidation of Acetone. Appl. Catal. A Gen..

[58] Wang X., Xie Y.C. (2001). The Promotion Effects of Ba on Manganese Oxide for CH_4_ Deep Oxidation. Catal. Lett..

[59] Stobbe E.R., de Boer B.A., Geus J.W. (1999). The Reduction and Oxidation Behaviour of Manganese Oxides. Catal. Today.

[60] Jo E.H., Chang H., Kim S.K., Choi J.-H., Park S.-R., Lee C.M., Jang H.D. (2016). One-Step Synthesis of Pt/Graphene Composites from Pt Acid Dissolved Ethanol via Microwave Plasma Spray Pyrolysis. Sci. Rep..

[61] Xie M., Chu T., Wang X., Li B., Yang D., Ming P., Zhang C. (2022). Effect of Mesoporous Carbon on Oxygen Reduction Reaction Activity as Cathode Catalyst Support for Proton Exchange Membrane Fuel Cell. Int. J. Hydrogen Energy.

[62] Minakshi M., Singh P., Issa T.B., Thurgate S., Marco R. (2004). De Lithium Insertion into Manganese Dioxide Electrode in MnO_2_/Zn Aqueous Battery. J. Power Sources.

[63] Ma R., Lin G., Zhou Y., Liu Q., Zhang T., Shan G., Yang M., Wang J. (2019). A Review of Oxygen Reduction Mechanisms for Metal-Free Carbon-Based Electrocatalysts. NPJ Comput. Mater..

[64] Ning X., Li Y., Ming J., Wang Q., Wang H., Cao Y., Peng F., Yang Y., Yu H. (2019). Electronic Synergism of Pyridinic- and Graphitic-Nitrogen on N-Doped Carbons for the Oxygen Reduction Reaction. Chem. Sci..

[65] Zhang S., Su W., Wei Y., Liu J., Li K. (2018). Mesoporous MnO_2_ Structured by Ultrathin Nanosheet as Electrocatalyst for Oxygen Reduction Reaction in Air-Cathode Microbial Fuel Cell. J. Power Sources.

[66] Khater D.Z., Amin R.S., Mahmoud M., El-Khatib K.M. (2022). Evaluation of Mixed Transition Metal (Co, Mn, and Cu) Oxide Electrocatalysts Anchored on Different Carbon Supports for Robust Oxygen Reduction Reaction in Neutral Media. RSC Adv..

[67] Ji X., Sun D., Zou W., Wang Z., Sun D. (2021). Ni/MnO_2_ Doping Pulping Lignin-Based Porous Carbon as Supercapacitors Electrode Materials. J. Alloys Compd..

[68] Wu Y., Liu S., Wang H., Wang X., Zhang X., Jin G. (2013). A Novel Solvothermal Synthesis of Mn_3_O_4_/Graphene Composites for Supercapacitors. Electrochim. Acta.

[69] Gautam R.K., Bhattacharjee H., Venkata Mohan S., Verma A. (2016). Nitrogen Doped Graphene Supported α-MnO_2_ Nanorods for Efficient ORR in a Microbial Fuel Cell. RSC Adv..

[70] Jaeel A.J. (2023). COD Reduction and Power Generation from Dual-Chamber Microbial Fuel Cells Fed with Sheep Manure Wastewater. Int. J. Ambient. Energy.

[71] Kim K., Takahashi T., Yumioka R., Hibino T. (2023). Sediment Microbial Fuel Cells in Oxidative Sedimentary Environments Using Iron Substrate as Voltage Booster. J. Power Sources.

