# Supplementary material for: Xerogel-Derived Manganese Oxide/N-Doped Carbon as a Non-Precious Metal-Based Oxygen Reduction Reaction Catalyst in Microbial Fuel Cells for Energy Conversion Applications
Source: Nanomaterials (Basel). 2023 Nov 15;13(22):2949. doi: 10.3390/nano13222949 (PMC10674280; doi:10.3390/nano13222949)
Supplement: Supplementary file 1 [file nanomaterials-13-02949-s001.zip › nanomaterials-2527929-supplementary.pdf]

Supporting Information

Xerogel-Derived Manganese Oxide/N-Doped Carbon as a Non-Precious Metal-Based Oxygen Reduction Reaction Catalyst in Microbial Fuel Cells for Energy Conversion Applications

Wu Hao, Sang-Hun Lee *, Shaik Gouse Peera *

Department of Environmental Science, Keimyung University, Daegu 42601, Republic of Korea

Corresponding authors:

Sang-Hun Lee (shlee73@kmu.ac.kr)
Shaik Gouse Peera (gouse@kmu.ac.kr)

S1. Physical and electrochemical characterizations

Particle size, graphitic nature of the Co/NCS and NCS catalysts are studied by using powder X-ray diffraction (XRD, Rigaku (D/Max-2500) diffractometer) analysis with Cu-Kα radiation source (λ = 1.5406 Å). The morphology of the Co/NCS and NCS catalysts are studied by scanning electron FESEM (Hitachi SU8220) and transmission electron microscope (FETEM, Titan G2 ChemiSTEM Cs Probe (FEI Company, The Netherlands). The oxidation state of cobalt and identification of different bonding of N and S are examined by X-ray photoelectron spectroscopy (XPS) using a Thermo Scientific K-Alpha X-ray photoelectron spectrometer.

S2. Electrochemical Characterizations of the N-CC, MnO/N-CC catalysts

Electrochemical studies of the N-CC, MnO/N-CC catalysts are studied in a traditional three electrode system with glassy carbon electrode (effective surface area of 0.1257 cm^2) as working electrode, graphite rod and saturated calomel electrode as counter and reference electrodes, respectively. The electrochemical studies were performed by using Biologic instruments SP-150e, potentiostat/galvanostat in 0.1 M $HClO_4$/0.1 M KOH/0.1 M PBS. For rotating disk studies, catalyst (N-CC, MnO/N-CC or Pt/C) ink is made by dispersing 4 mg of the catalyst in 1 mL of ethanol: water mixture (1:3) and the solution is ultrasonicated for 30 mins, to which a Nafion solution (5 wt.%) of 15 µl is added and further ultrasonicated for 30 mins. A 14 µl of the resultant catalyst ink is deposited in the glassy carbon electrode and then allowed it to dry at room temperature (catalyst loading: 560 µg cm^{-2}). For comparison, the Pt/C catalyst (10 wt%) with a catalyst loading of 110 µg cm^{-2} is also deposited on the GCE.

The ORR electrochemical tests were performed by cyclic voltammetry (CV) and linear sweep voltammetry (LSV). Before the electrochemical tests, the electrolyte 0.1 M $HClO_4$ /0.1 M KOH / 0.1 M PBS buffer solution is saturated with N_2/O_2 gas. The CV and LSV curves were recorded in the potential range of 0-1.2 V vs. RHE with a scan rate of 50 and 10 mV s^{-1}, respectively. For Koutechy-Levich (K–L) plots, the LSV curves were recorded at different rotations speeds from 800 to 2400 rpm. The number of electrons transferred in ORR process at different electrode potentials are calculated from the slopes of Koutechy-Levich (K-L) plots i.e plots of j^{-1} vs. $\omega^{-1/2}$ using the following questions.

$$\frac{1}{j} = \frac{1}{j_k} + \frac{1}{j_L} \tag{S1}$$

$$\frac{1}{j} = \frac{1}{j_k} + \frac{1}{B\omega^{1/2}} \tag{S2}$$

$$B = 0.62\, n\, F C_{O_2} {D_{O_2}}^{2/3} v^{-1/6} \tag{S3}$$

where j is measured current density, j_L is diffusion-limiting current density, j_k is kinetic current density, ω is angular velocity (rad s^{-1}). B is a parameter calculated from the equation (S3); “n” is number of electrons transfer per O_2 molecule, F is Faraday constant (96,485 C mol^{-1}), C_{O_2} is the concentration of oxygen in electrolyte 1.26×10^{-6} mol cm^{-3}, D_{O_2} is diffusion co-

efficient of oxygen in the solution $(\mathbf{1.93} \times \mathbf{10^{-5} cm^2 s^{-1}})$ and υ is kinematic viscosity of 0.1 M aqueous $HClO_4$ electrolyte (ν=0.001009 cm^2 s^{-1}. The number 0.62 is adopted when the rotation speed is expressed in rad s^{-1}. The stability of the Co/NCS and Pt/C catalysts have been carried out by potential cycling of the working electrode by recording repeated cyclic voltammograms between 0 to 1.0 V vs. RHE for 5000 and 10,000 cycles at the scan rate of 50 mV s^{-1}. To assess the degradation of the catalysts, the LSVs were recorded after stability test in O_2 saturated 0.1 M $HClO_4$ electrolyte with a scan rate of 10 mV sec^{-1}. All the potential was represented on relative hydrogen electrode (RHE) scale for convenience.

S3. Microbial fuel cell operation

The MFC reactor used was a dual chamber glass reactor with a volume capacity of 250 mL each (with a working volume of 200 mL). The carbon brush was used as an anode. The as received carbon brush was washed with ethanol and water to remove any dust and impurities. The two glass chambers are separated by a proton exchange membrane (Nafion 117). The as received membrane was pre-treated in 30wt% H_2O_2, then in 0.5 M H_2SO_4 and finally in deionized (DI) water. Each step is performed for 1 h at 80 °C. The pre-treated membrane is then stored in DI until use. The cathode used was a commercial GDL (geometric area = 2.6 × 5.5 cm). The cathode catalyst Co/NSC and Pt/C in the form of catalyst ink is deposited onto the GDL. The cathode catalyst ink is prepared by dispersing definite of the Co/NSC catalyst in isopropyl alcohol and ultrasonicated for 30 mins, to which 30 wt.% of Nafion (5 wt.%) solution is added and further ultrasonicated for 30 mins to obtain the final catalyst ink. The catalyst ink is then coated onto the GDL by using paint brush layer by layer until a desired catalyst loading 4 mg cm^{-2} for both MnO/N-CC catalysts catalyst while 0.5 mg cm^{-2} in case of Pt/C catalyst is achieved. The two MFC chambers were coupled with the Nafion 117 membrane, and two chambers are filled with 200 mL of the buffer solution (pH=7) on both sides. The anode chamber consists of 20 mM sodium acetate + 2M Glucose solution. The cathode is purged continuously with atmospheric air with the help of an air pump at a controlled flow rate (~10 ml min^{-1}). In this work, we have "utilized bacterial seed sample", from sewage water, collected from the local drainage as the source of bacteria. A sample of 10 mL sewage drainage water sample is added to the anodes of fresh MFC set up, allowing the bacteria to culture naturally in the anode chamber. Sewage water is a good source of mixed bacterial strains and mimics the most realistic wastewater treatment applications. In addition, as a source of carbon, we add 20 mM sodium acetate every alternative day to the anode chamber and allow the bacteria to grow. Over the course of time, bacteria attaches the carbon brush fibers and grow continuously, forming a biofilm. The biofilm formation can be assessed from the fact that the OCV slowly rises and reaches the maximum after a period of time. After reaching a steady and stable OCV, then the polarization curves were recorded. The anode and cathodes are connected to the potentiostat to monitor the open circuit voltage. The polarization curves were recorded by linear sweep voltammetry at a scan rate of 1 mV sec^{-1}. The current density and power densities were normalized according to the cathode geometric area and volume of the anolyte. For the TOC measurements, 5 mL of the anolyte is taken at a specified intervals and diluted to 30 mL with distilled water and the samples are stored in a refrigerator and later sent to the TOC

analysis. The MFC is operated with 100 Ω resister and an ample of solution from the anode cham-ber is collected at specified intervals and analyzed for TOC measurement

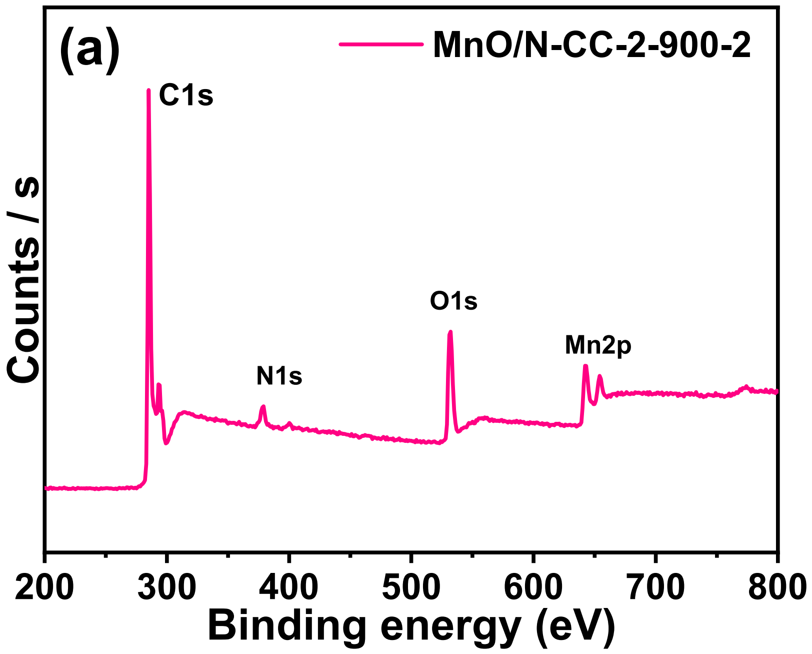

Figure. S1. XPS survey spectrum of MnO/N-CC-2-900-2 catalyst

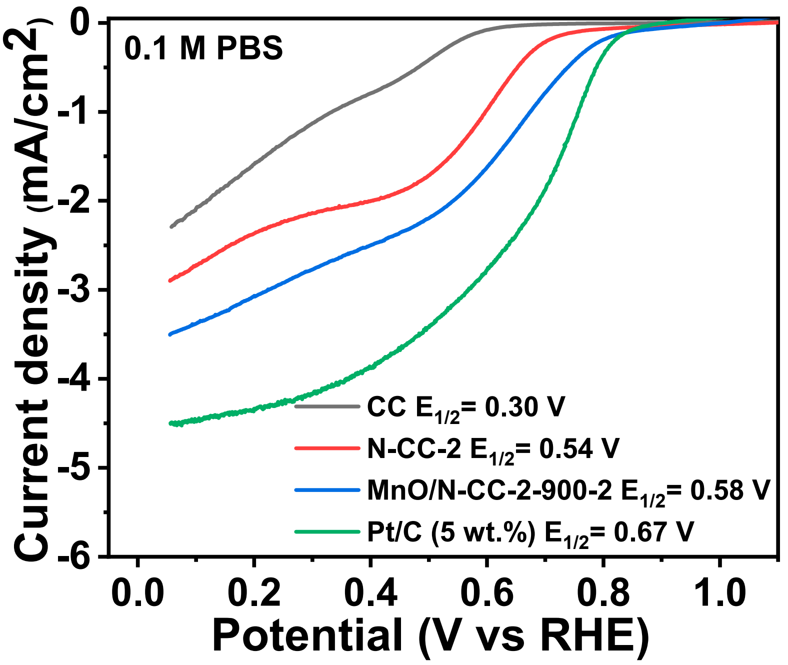

Figure. S2. LSV curves for CC, N-CC-2 and MnO/N-CC-2-900-2 and Pt/C catalyst in O_2 saturated 0.1 M phosphate buffer solution (PBS).

Figure. S2 shows that CC catalyst show very low ORR activity, whereas N-CC-2 shows significant shift in the onset potentials confirming the N-doping helps in enhancing the ORR activity in neutral media. Deposition of MnO nanoparticles on the N-CC-2 further shifts the ORR onset potentials positively, confirming that MnO deposition helps to further lower ORR overpotential. Similar to the ORR superiority of Pt/C in acidic media (Figure. 6e of the main article), Pt/C still holds better ORR kinetics also in neutral media. The half-wave potentials for CC, N-CC-2, MnO/N-CC-2-900-2 and Pt/C catalyst were found to be 0.30, 0.54, 0.58 and 0.67 V respectively. As per the new and re-performed ORR data on neutral media (as shown in Figure S2, above), suggest that the developed catalyst also show decent performance in the RDE studies, and the obtained results are similar to the MFC results (in RDE Pt/C > MnO/N-CC >N-CC) and in the MFC also follows a similar trend (Pt/C > MnO/N-CC >N-CC).

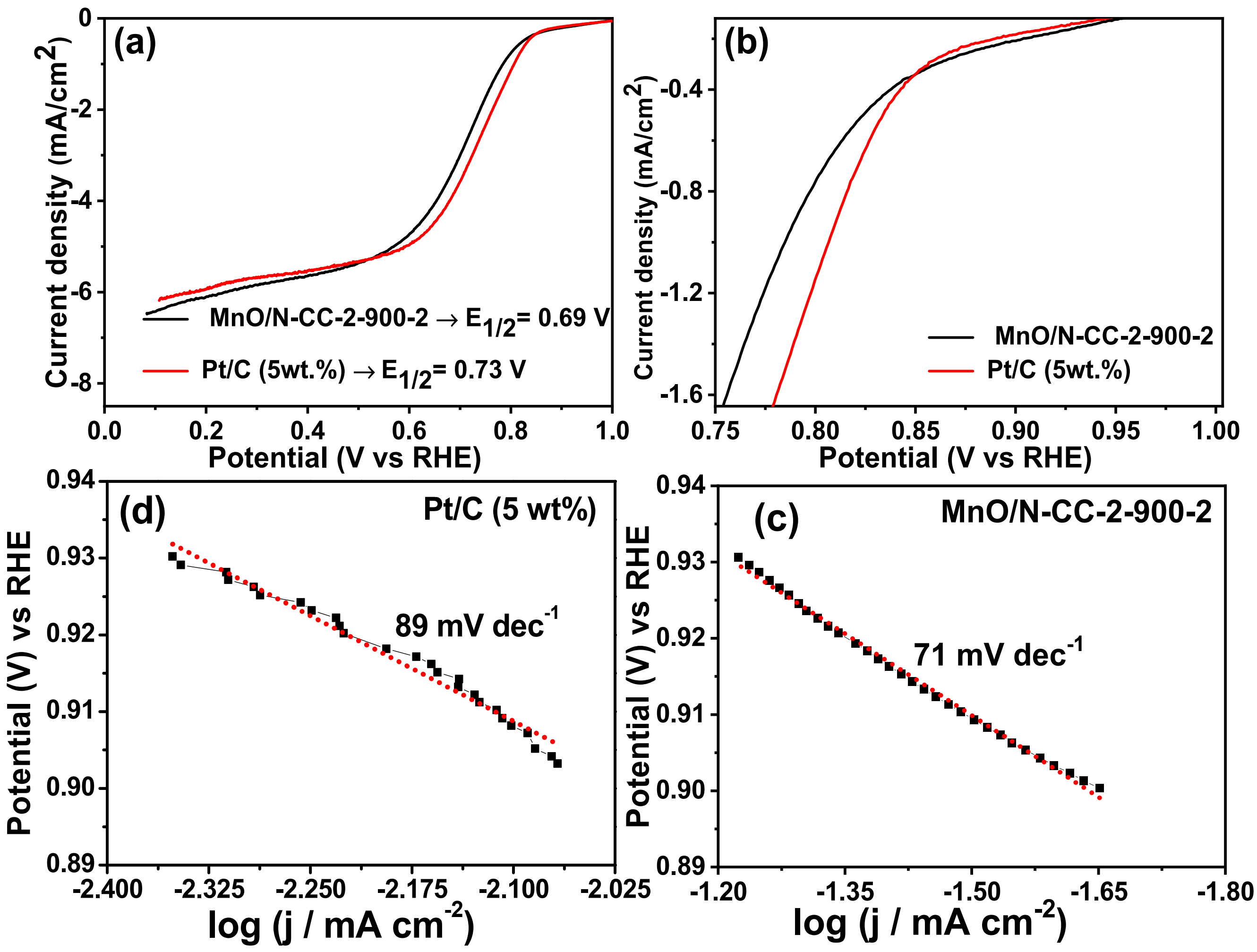

Figure. S3. (a) LSV curves MnO/N-CC-2-900-2 and Pt/C (5 wt.%) catalysts (b) LSV-zoomed portion representing kinetic region of MnO/N-CC-2-900-2 and Pt/C (5 wt.%) catalysts. Tafel plots for (c) MnO/N-CC-2-900-2 and (d) Pt/C (5 wt.%) catalysts.

Tafel slopes for MnO/N-CC-2-900-2 and Pt/C (5 wt.%) catalysts were found to be 71 mV and 89 mV dec^{-1} respectively. The lower slope values for MnO/N-CC-2-900-2 catalyst indicates that MnO/N-CC-2-900-2 catalyst has relatively faster ORR kinetics compared Pt/C, which can be attributed to the slightly improved kinetic region for MnO/N-CC-2-900-2 catalyst as seen from the Figure. S3b.

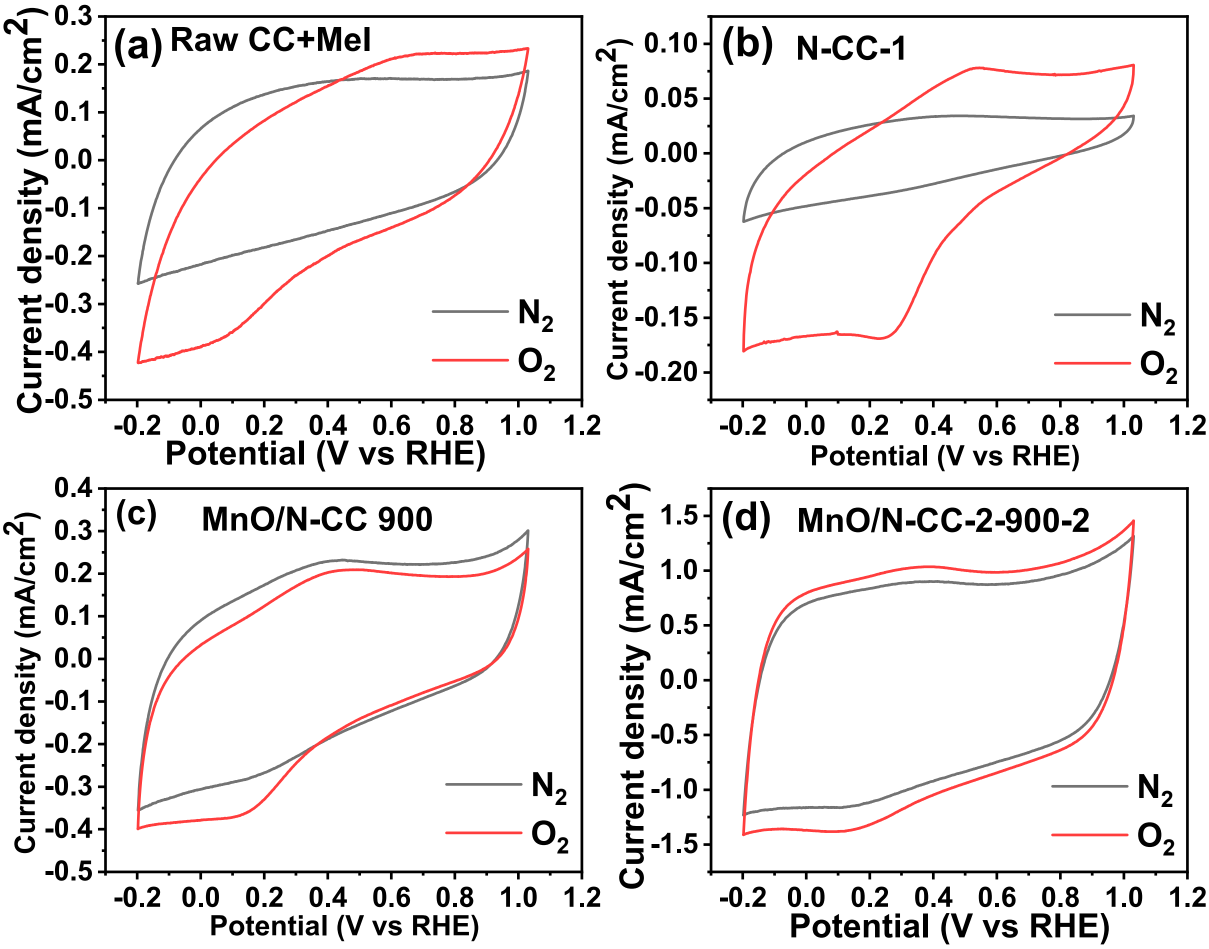

Figure. S4. CV curves for the optimized catalysts (a) Raw-CC+mel (b) N-CC-1 (c) MnO/N-CC-900 and (d) MnO/N-CC-2-900-2 recorded in 0.1 M $HClO_4$ solution saturated with O_2/N_2 gas at 25 °C. Scan rate 50 mV/sec.

CV curves recorded in the N_2 atmosphere shows no signs of ORR activity, whereas in the O_2 atmosphere all the catalyst showed an obvious ORR redox peak in the cathodic region, indicating that all the catalysts are active for ORR in acidic electrolytes as demonstrated in the LSV studies shown in the main manuscript Figure. 6.

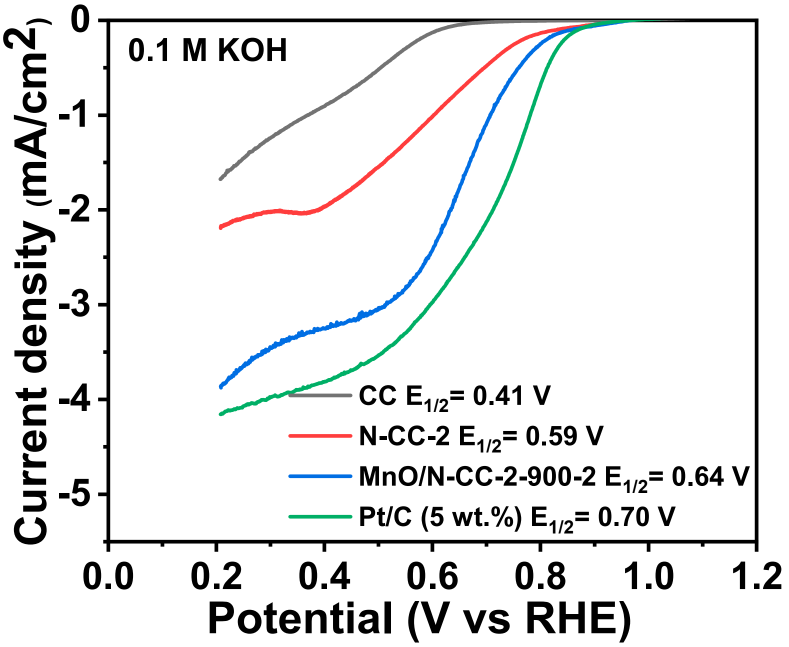

Figure. S5. LSV curves of the CC, N-CC-2, MnO/N-CC-2-900-2 and Pt/C catalysts 0.1 M KOH solution (base) electrolytes.

Figure. S5 shows that CC catalyst show very low ORR activity, whereas N-CC-2 shows significant shift in the onset potentials confirming the N-doping helps in enhancing the ORR activity in neutral media. Deposition of MnO nanoparticles on the N-CC-2 further shifts the ORR onset potentials positively, confirming that MnO deposition helps to further lower ORR overpotential. Similar to the ORR superiority of Pt/C in acidic media (Figure. 6e of the main article), Pt/C still holds better ORR kinetics also in alkaline media. The half-wave potentials for CC, N-CC-2, MnO/N-CC-2-900-2 and Pt/C catalyst were found to be 0.41, 0.59, 0.64 and 0.70 V respectively. As per the new and re-performed ORR data on neutral media (as shown in Figure S5, suggest that the developed catalyst also show decent performance in the RDE studies in alkaline electrolyte follows a trend of Pt/C > MnO/N-CC >N-CC.

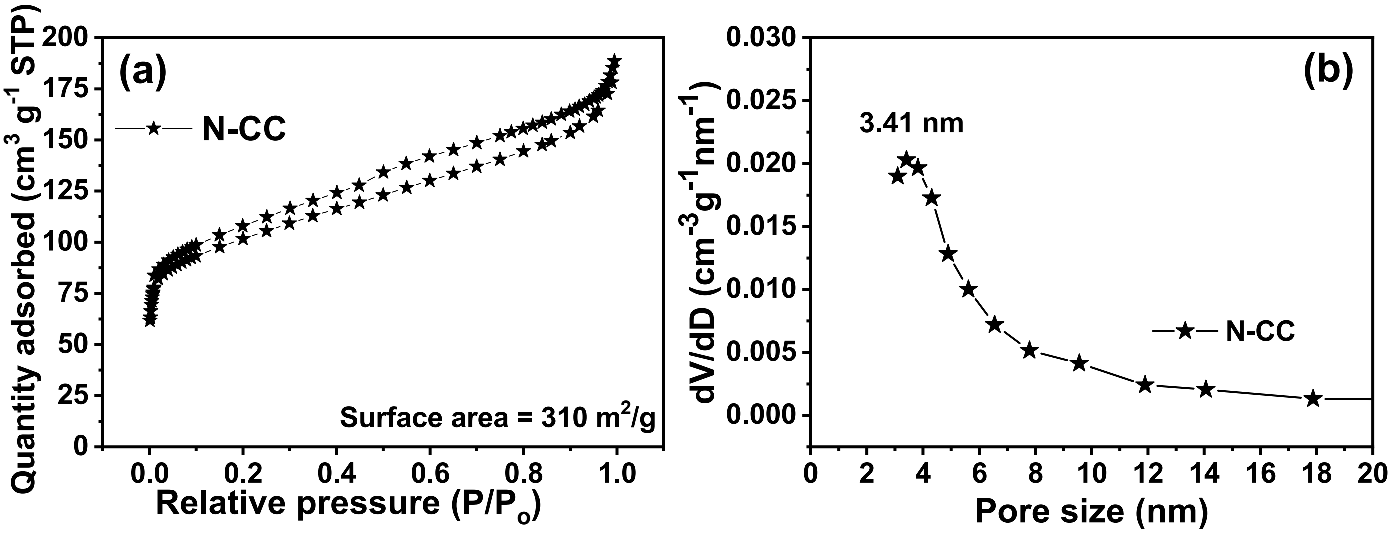

Figure. S6. (a) The N_2 adsorption/desorption isotherm of the N-CC-2 catalyst (b) pore size distribution curve of N-CC-2 catalyst.

The N-CC sample shows a typical adsorption/desorption isotherm of type IV, indicating that N-CC is mesoporous in nature, with a surface area of 310 m^2/g and pore size distribution analysis show that N-CC catalyst contains the abundant pores in the range of 3.41 nm. It is to be noted that MnO/N-CC catalyst surface area is 259 m^2/g, indicating that 84% of the surface area is still retained even after depositing the MnO nanoparticles.
